# Beyond quality control: biological roles of nonsense-mediated RNA decay

**DOI:** 10.1038/s44318-026-00794-0

**Published:** 2026-05-09

**Authors:** Kun Tan, Miles F Wilkinson

**Affiliations:** 1 Department of Obstetrics, Gynecology, and Reproductive Sciences, University of California San Diego, La Jolla, CA USA; 2 Institute of Genomic Medicine, University of California San Diego, La Jolla, CA USA

**Keywords:** Molecular Biology of Disease, RNA Biology

## Abstract

Nonsense-mediated RNA decay (NMD) was originally discovered by virtue of its “quality control” function of degrading aberrant mRNAs with premature termination codons (PTCs). NMD was subsequently found to be a highly selective and conserved RNA turnover pathway that also degrades subsets of normal mRNAs harboring stop codons in specific contexts. The discovery that many normal mRNAs encoding full-length normal proteins are degraded by NMD has led to a search for biological functions for NMD. In this review, we focus on the evidence for NMD’s roles in early embryonic development, nervous system development, spermatogenesis, thymic development, and other developmental processes in mice. NMD also has roles in stem cells, including dictating self-renewal vs. differentiation decisions in embryonic and neural stem cells. We also discuss evidence for NMD’s roles in some adult functions, such as circadian rhythm and neuronal activities. Finally, we highlight NMD’s causative roles in some human diseases and how therapeutic intervention of this critical pathway can be modeled in mice.

## Abstract (web summary)

Nonsense-mediated RNA decay shapes gene expression programs across development, stem cell fate, and adult physiology beyond its canonical quality control function.

## Graphical abstract

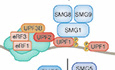

## Introduction

In the late 1970s, a new phenomenon was reported that ultimately became pertinent to a wide range of fields extending from molecular biology to developmental biology to human genetics. This new phenomenon was the ability of a disruption in the reading frame of an mRNA to elicit its rapid decay. In particular, it was discovered that introducing nonsense mutations into yeast (*Saccharomyces cerevisiae*) genes triggered rapid degradation of their corresponding mRNA products (Losson and Lacroute, 1979). Subsequent work began defining factors for this intriguing phenomenon (Leeds et al, 1991; Leeds et al, 1992) and later work established that the rapid mRNA decay elicited by nonsense mutations depends on a highly conserved biochemical pathway, present throughout the phylogenetic scale, now known as “nonsense-mediated RNA decay (NMD)” (Chang et al, 2007; Kishor et al, 2019; Kurosaki et al, 2019).

NMD degrades mRNAs harboring nonsense mutations because such mutations generate premature termination codons (PTCs)—a signal recognized by the NMD pathway. PTCs are also generated by frameshift mutations, and thus, frameshift mutations also trigger NMD. Both nonsense and frameshift mutations in disease genes are potentially dangerous, as they lead to the translation of truncated proteins that sometimes have deleterious dominant-negative activity. By stimulating the rapid decay of mRNAs transcribed from such disease genes, NMD reduces the expression of such truncated, sometimes harmful, proteins, and thus is protective (Chang et al, 2007; Frizzell et al, 2012; Thoren et al, 2010; Zheng et al, 2012).

Subsequent work led to the discovery that NMD is not only a quality control mechanism that promotes the decay of *aberrant* mRNAs, but it also promotes the decay of subsets of *normal* RNAs encoding normal full-length proteins (Chan et al, 2007; He et al, 2003; Lelivelt and Culbertson, 1999; Mendell et al, 2004; Moriarty et al, 1998). This intriguing discovery raised the possibility that NMD is not only a quality control pathway, but is also important for normal biological events. In support, scores of subsequent studies have provided evidence that NMD has roles in a wide range of fundamental processes (Jaffrey and Wilkinson, 2018; Kishor et al, 2019; Kurosaki et al, 2019; Nasif et al, 2018). NMD has also been strongly implicated in many human diseases, including cancer, as well as intellectual disability (ID) and neurodevelopmental disorders (Asthana et al, 2022; Jaffrey and Wilkinson, 2018; Nasif et al, 2018; Tan et al, 2022).

**Figure 1:**
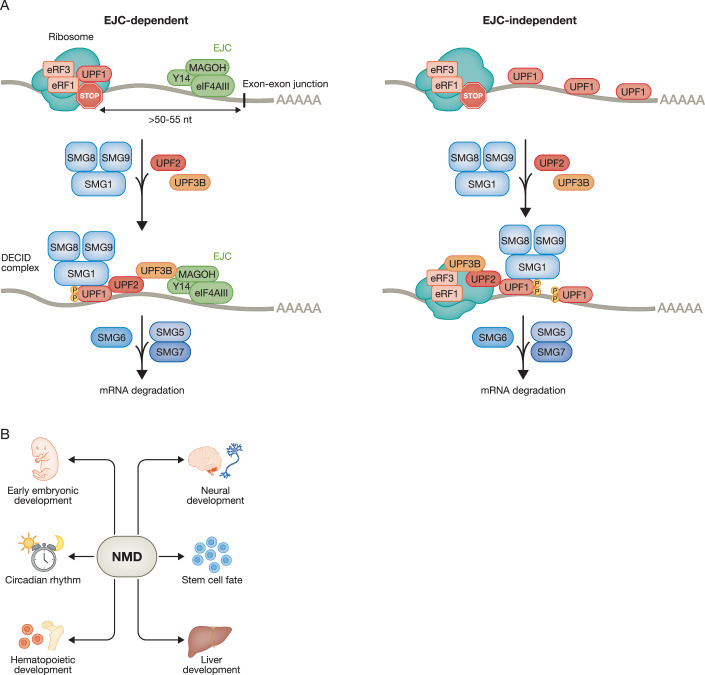
The NMD pathway. (**A**) RNAs with specific features are degraded rapidly by NMD. Left, one feature that triggers the NMD pathway involves the exon-junction complex (EJC), a set of proteins that is deposited just upstream of exon-exon junctions after RNA splicing. Exon-exon junctions >~55-nt downstream of the stop codon defining the main open reading frame (ORF) trigger NMD through this EJC-dependent mechanism. Right, another feature that can sometimes trigger NMD is a long 3’ untranslated region (UTR). While the exact mechanism is not known, it may be due to an excessive accumulation of NMD factors, including UPF1, on long 3’ UTRs. This subsequently leads to recruitment of factors that elicit NMD in an EJC-independent manner. Both mechanisms involve recruitment of the protein kinase, SMG1, which phosphorylates (p) UPF1, leading to recruitment of factors critical for RNA decay, including SMG5, SMG6, and SMG7. See text for more description. (**B**) Some biological roles of NMD in mammals.

NMD is a complex RNA decay pathway that utilizes over 20 proteins (Fig. 1A), including three key proteins—UPF1, UPF2, and UPF3—that are present in most eukaryotes and are considered to be core NMD factors (Chang et al, 2007; Kurosaki et al, 2019; Monaghan et al, 2023; Nasif et al, 2018). UPF1 is an RNA-binding protein recruited to all mRNAs that appears to remain stably bound to mRNAs destined to be degraded by the NMD pathway (Lee et al, 2015). UPF1 is an ATP-dependent RNA helicase that forms a NMD-promoting complex with a protein kinase dedicated to NMD, SMG1, as well as the translation termination factors eRF1 and eRF3 (Kashima et al, 2006; Kurosaki et al, 2019). UPF1 also interacts with UPF2, which, in turn, triggers UPF1 to undergo a conformational change that activates its RNA-helicase activity (Kurosaki and Maquat, 2016). UPF2 is also a scaffolding protein that forms a bridge between UPF1 and the adapter protein UPF3. Vertebrates express two isoforms of UPF3—UPF3A and UPF3B—from two different genes. UPF3A (also known as “UPF3”) is a very weak NMD factor in most cell types (with the possible exception of spermatocytes; see below), and thus there have been efforts to determine if UPF3A has other functions (Ma et al, 2019; Shum et al, 2016; Xie et al, 2023). UPF3B (also known as “UPF3X”) promotes the decay of a smaller number of NMD-target mRNAs than the core NMD factors, UPF1 and UPF2, suggesting that UPF3B only promotes the decay of a small subset of the mRNAs degraded by the NMD pathway as a whole (Chan et al, 2007; Yi et al, 2021). However, this notion was recently challenged by the finding that UPF3B and UPF2 target largely different mRNAs for decay in both embryonic stem cells (ESCs) and neural progenitor cells (NPCs) (Tan et al, 2025).

UPF3B interacts with the exon–junction complex (EJC), a NMD-promoting protein complex consisting of 3 factors: eIF4AIII, Y14 [RBM8A], and MAGOH (Fig. 1A). The EJC binds at exon–exon junctions formed after RNAs are spliced in the nucleus, and then it is transported with the spliced mRNAs to the cytoplasm, where the EJC functions in NMD as well as other cytoplasmic processes (Asthana et al, 2022; Le Hir et al, 2016; Woodward et al, 2017). An accessory protein that is often recruited to the EJC in the cytoplasm is CASC3, which drives an alternative branch of the NMD pathway (Cho et al, 2022). EJC proteins can serve as entry points for assembling distinct NMD-activating ribonucleoprotein complexes. For example, Y14, MAGOH, and eIF4AIII can activate NMD in a UPF2-independent manner, whereas RNPS1-dependent NMD appears to require UPF2 (Gehring et al, 2005; Mabin et al, 2018). In turn, the presence or absence of the EJC has an important role in determining which specific RNAs are degraded by NMD (Box 1).

The study of NMD in different species has been instrumental in uncovering the biological roles of this RNA decay pathway. For example, studies in *Saccharomyces cerevisiae* have shown that this organism has an active NMD pathway that is critical for growth on non-fermentable carbon sources (Murtha et al, 2019). In *Caenorhabditis elegans*, NMD has roles in mating. NMD-deficient *C. elegans* (harboring mutations in *smg genes*) have morphogenic defects in the male bursa and hermaphrodite vulva (Cali et al, 1999; Hodgkin et al, 1989). Interestingly, NMD has also been shown to influence *C. elegans* lifespan under some circumstances. Loss of NMD (as a result of knockout or knockdown of the *smg-2* gene) substantially shortens the extended lifespan elicited by simulated starvation (via genetic inhibition of the *daf-2/insulin/IGF-1* receptor pathway) (Son et al, 2017). Given that NMD activity declines during *C. elegans* aging (Son et al, 2017), these results have potential implications for the effects of diet and aging in humans. In *Drosophila (D.) melanogaster*, NMD appears to be critical for embryonic development. Analysis of NMD-deficient mutant *D. melanogaster* demonstrated that UPF1 and UPF2 (but not UPF3 or SMG1) are required to prevent widespread apoptosis during larval development (Avery et al, 2011; Metzstein and Krasnow, 2006). Mutations in NMD genes in flies also cause neuromuscular junction synapse defects (Long et al, 2010). Like flies, zebrafish (*Danio rerio*) require NMD for embryonic development. Morpholino-knockdown experiments in *D. rerio* showed that NMD factors are critical for embryo viability and embryonic development (Wittkopp et al, 2009). Defects include brain patterning abnormalities (mainly in the mid- to hind-brain boundary) and misregulation of transcripts important for differentiation and morphogenesis.

In this review, we focus on NMD’s roles in mammalian organisms (Fig. 1B). Most of the progress towards understanding the role of NMD in mammals in vivo has been made in mice, so we highlight these studies. Because the function of NMD has also been studied in some detail in stem cells, we also review studies on this topic, including those conducted in human stem cells. Our review provides an update on topics covered by previously published excellent reviews on the topic of biological roles of NMD (Han et al, 2018; Hwang and Maquat, 2011; Jaffrey and Wilkinson, 2018; Nasif et al, 2018).

Box 1 NMD-inducing signals Early studies demonstrated that one signal that triggers RNA decay by NMD is the presence of an exon–exon junction downstream of the stop codon defining the end of the main ORF (Carter et al, 1996; Nagy and Maquat, 1998; Thermann et al, 1998). Later studies demonstrated that the molecular entity responsible for this “dEJ rule” is the EJC (Chang et al, 2007; Kishor et al, 2019; Kurosaki et al, 2019). Given that EJCs are generally recruited to all exon–exon junctions, why do only exon–exon junctions downstream of the stop codons trigger NMD? The likely answer comes from a study that demonstrated that EJC protein, Y14, is displaced from RNAs by translocating ribosomes after RNAs are transported into the cytoplasm (Dostie and Dreyfuss, 2002). Thus, mRNAs with exon–exon junctions only in the coding region will be stripped of all EJCs by translocating ribosomes and thus avoid NMD. In contrast, RNAs that have one or more exon–exon junctions after the stop codon will retain EJCs since ribosomes cease translocating after reaching a stop codon. This scenario leads to a model in which mRNAs with at least one exon–exon junction downstream of the main ORF will harbor an NMD-promoting EJC complex for an extended period of time and thus are primed to undergo decay through NMD. In support, introduction of exon–exon junctions downstream of ORFs has been shown to trigger NMD (Carter et al, 1996; Nagy and Maquat, 1998; Thermann et al, 1998), and a large fraction of NMD-target mRNAs have at least one exon–exon junction downstream of the main ORF (Kurosaki and Maquat, 2016; Nagy and Maquat, 1998). An exception to this dEJ rule is the "−55 nucleotide (nt) boundary rule," a rule based on the finding that RNAs with the main ORF stop codon less than ~55 nt upstream of the last exon–exon junction often escape NMD (Nagy and Maquat, 1998). A likely explanation for this −55 nt boundary rule is that stop codons at such positions allow translocating ribosomes to collide with the EJC just upstream of the last exon–exon junction and thus displace this EJC from the RNA (given the known EJC “footpad” size and the fact that the EJC tends to be centered ~20–24 nt upstream of exon–exon junctions (Le Hir et al, 2016)).

There are also non-EJC-dependent mechanisms that degrade RNAs by the NMD pathway. For example, long 3’ untranslated regions (UTRs) and short ORFs upstream of the main ORF (uORFs) can trigger NMD (Kishor et al, 2019; Kurosaki et al, 2019; Munoz et al, 2023). While both long 3’UTRs and uORFs *can* elicit NMD, they do not necessarily do so. No specific 3’UTR length has been shown to elicit NMD (Toma et al, 2015). Half of mammalian mRNAs contain uORFs, and thus most uORFs must *not* be capable of eliciting NMD. Even an exon–exon junction downstream of a stop codon—a dEJ—will not necessarily elicit NMD; this depends on biological context (Tan et al, 2025).

## Roles of NMD in mice

Studies in NMD-deficient mice have provided evidence that NMD is critical for several different developmental processes in vivo, including early embryonic development, neural development, spermatogenesis, and hematopoietic stem and progenitor cell development (Fig. 1B). Below, we discuss the evidence for NMD’s roles in these developmental processes. We also discuss the evidence that NMD is important for adult functions. We also describe a mouse model that permits inducible disruption of NMD in mice (Echols et al, 2020). This inducible NMD-knockdown mouse model has the potential to elucidate stage-specific biological roles of NMD, as well as to determine NMD’s potential as a therapeutic target for treating human diseases.

### Early embryonic development

**Figure 2:**
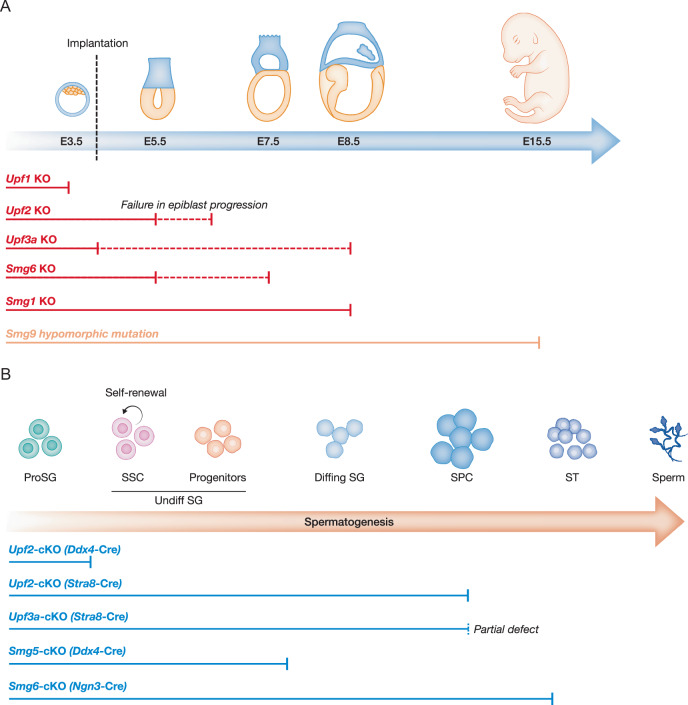
NMD perturbation causes arrest at different stages of early embryonic development and germ-cell development. (**A**) Global knockout (KO) of NMD factor genes causes embryonic lethality at the indicated embryonic (E) time points in mice. The time point of initial defect is indicated by the leftmost tick marks and lethality is indicated by tick marks after the dotted line. (**B**) Conditional (c) KO of NMD factor genes specifically in germ cells causes spermatogenic defects in mice. The cKO is mediated by the indicated Cre-driver mice, which delete floxed genes at different germ cell stages. The tick mark indicates the germ-cell stage at which major defects occur, such as developmental arrest or apoptosis (see text for details). Spermatogenesis is first initiated from male germ cell precursors called “prospermatogonia (ProSG).” ProSG give rise to spermatogonial stem cells (SSCs), which later differentiate to form spermatogonial progenitors, which then further differentiate to form spermatocytes (SPCs). The latter undergo meiosis to generate haploid cells called round spermatids (STs). These round STs differentiate into elongated STs that then transition through several further differentiation steps to ultimately become sperm (Tan and Wilkinson, 2023).

Global KO of any of several different NMD genes in mice causes early embryonic lethality, implying a role for NMD in early embryonic development. Interestingly, NMD-deficient mice embryos have been reported to have somewhat different phenotypes depending on the specific NMD gene mutated (Fig. 2A). *Upf1*-KO embryo die during the pre-implantation period (embryonic [E] day 3.5) (Medghalchi et al, 2001), *Upf2*-KO embryos die during the post-implantation period (E5.5–6.5) (Chousal et al, 2022), *Smg6*-KO embryos implant but then exhibit growth retardation at E7.5 (Li et al, 2015), and *Smg1*-KO mice embryos implant and gastrulate normally but then undergo developmental arrest at E8.5 and have several developmental abnormalities, including defects in the heart field and optic pit indentation (McIlwain et al, 2010).

Chousal et al recently performed an in-depth analysis of the early embryonic lethality caused by NMD deficiency (Chousal et al, 2022). This study observed that inner cell mass (ICM) cells in *Upf2*-null blastocysts rapidly regressed during in vitro outgrowth and were unable to give rise to embryonic stem cells (ESCs) in vitro. This ICM defect was specific, as *Upf2*-null trophectoderm cells undergo normal outgrowth in vitro. Staining *Upf2*-null blastocysts with antibodies against lineage-specific marker proteins indicated that NMD is specifically required for the progression of the pluripotent epiblast in vivo.

A change in NMD magnitude (strength) is a potentially powerful means by which NMD can influence biological processes. This follows from the fact that a shift in NMD magnitude will simultaneously change the stability (and hence the level) of batteries of NMD’s target transcripts. Chousal et al examined whether the magnitude of NMD is modulated during early embryonic development. They found that NMD is upregulated in the epiblast lineage during peri-implantation development (between E4.5 and 5.5). The increased NMD magnitude is accompanied by a decrease in the level of NMD-target mRNAs that are candidates to normally repress epiblast progression. Several NMD factors are upregulated precisely when NMD magnitude is upregulated. This raises the possibility that this increase in the level of NMD factors is responsible for the developmentally regulated increase in NMD magnitude and consequent reduction in the level of key transcripts during early embryonic development.

The finding that global KO of any of a number of different NMD factor genes causes early embryonic lethality strongly suggests that NMD has critical roles in early embryonic development. However, clouding this simple conclusion is the reported differences in phenotypes and lethality timing caused by loss of different NMD factors (Fig. 2A). Some of these differences could be more perceived than real; e.g., they could stem from differences in experimental approaches used by different investigators and/or variation in NMD factor disruption efficiency; e.g., a hypomorphic allele masquerading as a null allele. Indeed, a recent study generated mice with a hypomorphic mutation in the NMD factor gene, *Smg9*, and found that these *Smg9*-mutant mice did not display obvious defects until E15.5 (Shaheen et al, 2016) - much later than the NMD-deficient mice described above (Chousal et al, 2022; Li et al, 2015; McIlwain et al, 2010; Medghalchi et al, 2001). These *Smg9*-homozygous-mutant embryos exhibited a range of variable and incompletely penetrant phenotypes, including hemorrhage and exencephaly.

A non-mutually exclusive possibility is that some of the reported defects in NMD factor-deficient mice are not caused by NMD deficiency but instead caused by perturbed *non*-NMD functions. In support of this possibility, the NMD factors, UPF1, UPF2, SMG1, SMG6, and SMG7 are known to be involved in *non*-NMD functions, including DNA damage responses and telomere integrity maintenance (Han et al, 2018; Hwang and Maquat, 2011) (Table 1). Both functions are known to maintain cell viability, and thus defects in these pathways could potentially contribute to the embryonic defects in mice deficient in NMD factors.

**Table 1:** Non-NMD functions of NMD factors.

NMD factors	Non-NMD functions
UPF1	Staufen (STAU)1- mediated mRNA decay DNA damage responses Histone mRNA decay (at the end of S phase) DNA replication Telomere maintenance Small RNA-induced mRNA downregulation
UPF2	DNA damage response Telomere maintenance
UPF3A	Transcriptional adaptation
UPF3B	
SMG1	DNA-damage responses Telomere maintenance
SMG5	Telomere maintenance
SMG6	DNA damage responses Telomere maintenance
SMG7	DNA damage responses Telomere maintenance

The NMD factor, UPF3A, is a good candidate to influence embryonic development through *non*-NMD biochemical functions. Both gain- and loss-of-function experiments have demonstrated that, under most circumstances, UPF3A is an extremely weak NMD factor that primarily acts only when UPF3B is mutated or knocked down (Chan et al, 2007; Lykke-Andersen et al, 2000; Shum et al, 2016; Wallmeroth et al, 2022; Yi et al, 2022). This issue was recently studied in detail by Chen et al, who showed that UPF3A is dispensable for NMD in several adult mouse tissues, as well as ESCs and primary cells generated from a *Upf3a* conditional-KO mice (based on analysis of 33 NMD-target mRNAs) (Chen et al, 2023). Given that UPF3A is a weak and/or dispensable NMD factor, investigators have searched for other biochemical functions for UPF3A. For example, investigators have obtained evidence that UPF3A can act as a global NMD repressor in some cell types (Shum et al, 2016) but not in immortalized or malignant cells (Wallmeroth et al, 2022; Yi et al, 2022). In zebrafish, UPF3A has been shown to support the “transcriptional adaptation” response that upregulates paralogs of some genes harboring frame-disrupting mutations (Ma et al, 2019; Xie et al, 2023). It remains to be determined if UPF3A functions in the transcriptional adaptation response in mammals. It will be important to decipher how UPF3A functions biochemically, as it is clearly important biologically. For example, UPF3A has been shown to be critical for early embryonic development, as mice with a null mutation in *Upf3a* die between E4.5 and E8.5, with morphological defects observed as early as E3.5 (Shum et al, 2016). UPF3A is also important for spermatogenesis, as described below.

In addition to being knocked out globally, some NMD factor genes have been conditionally knocked out in specific biological contexts. In some cases, this leads to lethality. For example, conditional loss of UPF2 in the developing liver (through breeding *Upf2-*floxed mice with *Alfp*-Cre mice) leads to failure of hepatocyte terminal differentiation coupled with perinatal lethality (Thoren et al, 2010). Induced loss of UPF2 within the hematopoietic cell lineage (by injecting *Upf2*^fl/fl^;*Mx1*-Cre mice with the *Mx1* promoter activator polyinosinic-polycytidylic acid) leads to rapid loss of almost all nucleated cells within the bone marrow, followed by lethality (Weischenfeldt et al, 2008).

The underlying molecular mechanisms by which NMD governs early embryonic development in mice remain largely unclear. Studies in other organisms have shown that NMD influences biological events by degrading key NMD-target mRNAs. For example, rescue experiments have demonstrated that NMD is required for *D. melanogaster* embryonic survival because NMD degrades the mRNA encoding the pro-apoptotic protein GADD45 (Nelson et al, 2016). Likewise, rescue experiments in cultured mammalian cells have identified key target mRNAs that NMD acts on to drive several other biological events, including the unfolded protein response and neural differentiation (Karam et al, 2015; Lou et al, 2014). To identify NMD-target mRNAs in early mouse embryonic development, Chousal et al performed RNA sequencing (RNAseq) analysis on E3.25 embryos from *Upf2*-null and control mice (Chousal et al, 2022). Many of the upregulated genes they identified have known NMD-inducing features and thus are likely to encode NMD-target mRNAs in the early embryo. Enriched among these NMD targets are functions related to cell-cycle arrest and apoptosis, raising the possibility that NMD acts in early mouse embryogenesis by promoting cell proliferation and/or cell survival. Indeed, it is known that NMD promotes cell proliferation and prevents programmed cell death in other biological contexts and in species in addition to mice (Avery et al, 2011; Lou et al, 2014; Nelson et al, 2016; Weischenfeldt et al, 2008).

### The nervous system

The initial discovery that fueled the notion that NMD is important for the nervous system was the strong association of debilitating mutations in the NMD gene, *UPF3B*, with ID (Tarpey et al, 2007). Pedigree analysis by this study and subsequent studies (Addington et al, 2011; Laumonnier et al, 2010; Lynch et al, 2012; Xu et al, 2013) demonstrated causality. Individuals with *UPF3B* mutations also commonly have neurodevelopmental disorders, including autism, attention-deficit/hyperactivity disorder, developmental delay, major communication deficits, and schizophrenia (Nguyen et al, 2014; Tarpey et al, 2007). Some individuals with loss-of-function *UPF3B* mutations almost completely lack speech and language skills (Domingo et al, 2020; Tejada et al, 2019).

To define the underlying mechanism, mice with a null mutation in the *Upf3b* gene were generated (Huang et al, 2011). These NMD-deficient mice suffer from specific learning and memory deficits, including fear-conditioned learning, and thus may replicate some aspects of the behavioral defects in UPF3B-deficient humans (Huang et al, 2018b). In part, these behavioral defects may stem from abnormal neural connectivity, as cortical pyramidal neurons in *Upf3b*-KO mice exhibit impaired dendritic spine maturation in vivo (Huang et al, 2018b). In addition, UPF3B has been found to be critical for olfactory neural development, as *Upf3b*-KO mice exhibit altered frequencies of specific olfactory sensory neuron (OSN) subsets and exhibit changes in the *Olfr* gene repertoire (Tan et al, 2020).

As a first step towards understanding how UPF3B molecularly functions in the nervous system, UPF3B-regulated transcripts have been identified in both the frontal cortex (Huang et al, 2018b) and mature OSNs (Tan et al, 2020). In these studies, high-confidence NMD-target mRNAs were identified based on (i) their being upregulated upon UPF3B loss and (ii) their containing at least one well-established NMD-inducing signal. Interestingly, several of these NMD targets encode proteins involved in neural development and cell differentiation. Recently, Tan et al identified high-confidence UPF3B-dependent NMD-target mRNAs from two distinct brain regions in adult mice—the frontal cortex and the cerebellum—and, surprisingly, found only modest overlap between the NMD-target mRNAs in these two regions, even among RNAs co-expressed in these brain regions (Tan et al, 2025). This unexpected finding suggests that NMD-target mRNAs can be highly context-dependent, varying across brain regions and possibly different neuronal cell types. This may contribute to the distinct biological functions of NMD in different parts of the brain.

As another approach to examine the role of NMD in the nervous system, Lin et al conditionally knocked out the NMD gene, *Upf2*, specifically in neural progenitors (using *Emx1*-Cre and *Nestin*-Cre mice) (Lin et al, 2024). This conditional KO led to reduced proliferation of radial glia progenitor cells coupled with their precocious transition into intermediate progenitors, ultimately leading to reduced upper-layer neurons and microcephaly. A CRISPRi rescue screen identified TRP53 perturbation as being the most efficient at rescuing this NPC proliferation defect in vitro. This p53 pathway-dependent rescue was also observed in vivo, as neural progenitors proliferated more in *Trp53*/*Upf2* double-KO mice than in *Upf2*-cKO mice. TRP53 loss also partially rescued the microcephaly of *Upf2*-cKO mice. The NMD and p53 pathways may have an intimate molecular relationship in this developmental setting, as the authors found that NMD promotes neural proliferation by degrading the mRNA transcribed from the TRP53-dependent gene encoding the cell-cycle inhibitor protein CDKN1A.

Given that the EJC is also critical for NMD (see Introduction), this predicts that mice deficient for EJC components will also have neural defects. Indeed, mice lacking *only a single copy* of any of the 3 core (“constitutive”) EJC genes (*Rbm8a*, *Magoh*, or *Eif4a3*) have severe neural defects (Mao et al, 2016; Mao et al, 2015; Silver et al, 2010). These *Rbm8a-*, *Magoh-*, and *Eif4a3-*heterozygous mice also suffer from microcephaly, just as the *Upf2*-cKO mice described above (Lin et al, 2024). In striking contrast to these neural defects in mice lacking one copy of these EJC genes, mice lacking a single copy of NMD factor genes exhibit no discernable phenotype (Li et al, 2015; McIlwain et al, 2010; Medghalchi et al, 2001; Shaheen et al, 2016; Weischenfeldt et al, 2008). A likely explanation is the fact that, in addition to NMD, the EJC has other roles, including in RNA splicing, nuclear RNA export, translation, and cytoplasmic RNA localization (Asthana et al, 2022; Le Hir et al, 2016; Woodward et al, 2017).

In this regard, it is worth noting that KO of the gene encoding the non-constitutive EJC subunit—CASC3—causes much less severe brain defects than KO of genes encoding the constitutive EJC components RMB8A, MAGOH, and EIF4A3 (Mao et al, 2017). Loss of CASC3 causes neural developmental delay but otherwise CASC3 was found to be largely dispensable for brain development. It will be important in the future to determine whether this is because CASC3 differs from other EJC components in being primarily recruited to the EJC in the cytoplasm rather than in the nucleus (Mabin et al, 2018). While one of CASC3’s functions is in NMD (Mabin et al, 2018), it also influences translation and cytoplasmic RNA localization (Chazal et al, 2013; Macchi et al, 2003; van Eeden et al, 2001).

NMD also has roles in *mature* neurons. For example, evidence suggests that NMD functions in neuronal axons, the neuronal projections that send signals to other neurons. Colak et al found that conditional KO of the *Upf2* gene in dorsal commissural neurons disrupts the normal trajectory of their axon after crossing the spinal cord ventral midline (Colak et al, 2013). While the underlying mechanism by which NMD influences the trajectory of dorsal commissural neuron axons was not definitively determined, Colak et al identified a good candidate to be involved. They found that the mRNA encoding the axon guidance protein, ROBO3.2, is a NMD target. Intriguingly, its degradation by NMD only occurs after the commissural neuron axons have crossed the ventral midline. This rapid decay occurs precisely when this mRNA begins translating the ROBO3.2 protein, which is consistent with the considerable evidence that activation of the NMD pathway depends on translation (Belgrader et al, 1993; Carter et al, 1995). While it is seemingly paradoxical that an mRNA would be induced to simultaneously undergo translation and rapid decay, in fact, it is a rational approach to achieve transient expression of a protein. Indeed, this translation/mRNA decay-coupled mechanism may be a general approach used by cells when they need to express proteins in a transient manner in vivo.

A follow-up study—Notaras et al (Notaras et al, 2020)—obtained evidence that NMD also functions in another neuronal projection—the dendrite—the branched process extending from the cell body that is responsible for receiving signals from other neurons. This study was initiated by the discovery that KO of the NMD gene, *Upf2*, in postmitotic glutamatergic neurons (via breeding with inducible *Camk2α*-CreERT2 mice) leads to repetitive behavior coupled with perturbed learning, memory, synaptic plasticity, and spine density in adult mice. To investigate the underlying mechanism, these investigators turned to studying UPF2-deficient hippocampal neurons in vitro. This analysis revealed that UPF2 promotes expression of the excitatory neurotransmitter receptor, Glutamate Receptor 1 (GLUR1), by promoting its translation specifically in dendrites—the subcellular region where GLUR1 functions. These authors found that UPF2 also drives GLUR1 expression by another mechanism - it inhibits GLUR1 protein internalization, thereby promoting dendritic cell-surface expression. As evidence that UPF2 acts at the local level, these investigators found that UPF2, as well as other NMD factors, are concentrated in dendrites. Through “rescue and mimic” experiments in which NMD factors were selectively knocked down in neuronal synaptic channels, they found that NMD acts through the NMD-target mRNAs, *Prkag3* and *Arc* mRNA, to increase GLUR1 expression on the surface of the dendrites. To investigate in vivo significance, Notaras et al performed rescue experiments in adult mice and obtained evidence that UPF2 also acts through *Prkag3* and *Arc* mRNA regulation to increase hippocampal spine density in vivo. Finally, they found that local regulation of surface GLUR1 expression was not restricted to UPF2, as knockdown of the NMD factor, UPF3B, specifically in synaptic channels, also modulated GLUR1 expression. Together, the experiments in Notaras et al provide compelling evidence that NMD functions at the subcellular level—in dendrites—to influence neuron function.

Using a similar genetic strategy, Johnson et al knocked out *Upf2* by breeding with non-inducible *Camk2α*-Cre mice and found that these mutant mice (like the inducible cKO generated by Notaras et al described above) exhibit memory deficits (Johnson et al, 2019). In addition, these constitutive NMD-deficient mice suffered from social and communication deficits. Consistent with the growing evidence that neural dysfunction can be caused by inflammation, these *Upf2*-cKO mice also suffered from inflammation, coupled with elevated expression of immune genes, in the forebrain. The synaptic and behavioral deficits in these mutant mice were partially rescued by treatment with two FDA-approved anti-inflammatory drugs, raising the possibility that these *Upf2*-cKO mice could be a useful mouse model for treating neural diseases linked to inflammatory responses.

NMD also has roles in the nervous system in humans. As described above, pedigree analysis of numerous families has demonstrated that both nonsense and missense mutations in *UPF3B* cause ID and are associated with autism, schizophrenia, and/or attention-deficit/hyperactivity disorder in humans (Nguyen et al, 2014; Tarpey et al, 2007). In addition, it has been reported that copy number variation in several NMD genes—including *UPF2, UPF3A, UPF3B, EIF4A3, RNPS1*, and *SMG6*—are significantly associated with ID, neurodevelopmental disorders and brain malformations (Jaffrey and Wilkinson, 2018; Nguyen et al, 2013). Mutations in EJC genes are also associated with neurological disease. For example, deletions in 1q21.1, which include the gene encoding the EJC core component, RBM8A, are associated with increased incidence of intellectual disability, epilepsy, autism spectrum disorder, and schizophrenia (Brunetti-Pierri et al, 2008; Mefford et al, 2008).

### Spermatogenesis

Spermatogenesis is a highly orchestrated developmental process that involves male germ cells undergoing proliferation and meiosis, followed by differentiation into sperm (Fig. 2B). The NMD factors, UPF1, UPF2, and SMG6, have been found to be highly expressed in the post-meiotic stage of male germ cells—spermatids. Interestingly, these NMD factors are concentrated in germ cell-specific cytoplasmic (perinuclear) structures called chromatoid bodies (CBs) (Bao et al, 2016; Fanourgakis et al, 2016; Lehtiniemi et al, 2022). This raised the possibility that NMD is critical for this stage of male germ cell development. In support, Lehtiniemi et al showed that mice that have lost the NMD factor, SMG6, at the early postnatal spermatogonia stage (via breeding floxed-*Smg6* mice with *Ngn3*-Cre mice) have a complete arrest of spermatogenesis at the early spermatid stage (Lehtiniemi et al, 2022) (Fig. 2B). These germ cell-specific *Smg6*-cKO mice also exhibit extensive transcriptome misregulation, including a failure to eliminate meiotically-expressed transcripts in early haploid cells. This suggests that NMD drives germ-cell progression by shaping the transcriptome in a developmentally progressive manner.

More evidence for a role of NMD in spermatids comes from Fanourgakis et al, who found that a CB-enriched protein in spermatids—TDRD6—is essential for the CB localization of UPF1 and the interaction of UPF1 with UPF2 (Fanourgakis et al, 2016). *Tdrd6*-null male germ cells exhibit disrupted CB formation and developmental arrest at the round spermatid stage (Vasileva et al, 2009).

To examine the possibility of earlier developmental roles of NMD, Bao et al disrupted NMD in prospermatogonia (ProSG) and spermatogonia by breeding floxed-*Upf2* mice with *Ddx4*-Cre and *Stra8*-Cre mice, respectively (Bao et al, 2016). They found that adult *Upf2*-cKO mice that ablate *Upf2* at the ProSG stage—the transient germ-cell stage occurring between ~E12.5 and ~postnatal day (P)2 (Fig. 2B)—are infertile and almost completely lack germ cells in their testes (referred to as “Sertoli-only syndrome” in humans). Germ-cell loss was observed as early as during the transition between the ProSG and spermatogonia stages (P3), and histological defects (including further germ cell loss) were observed as early as the beginning of meiosis (P10). They found that adult *Upf2*-cKO mice that ablate *Upf2* at the later stage—spermatogonia (Fig. 2B)—are also infertile and contain few post-meiotic germ cells. Defects were first observed at the pachytene stage of meiosis (P14), including signs of delayed meiotic entry. These cKO mice initially exhibited extensive loss of spermatocytes and, later, a loss of spermatids.

Together, the phenotypes of the three NMD-deficient cKO mice strains studied by Lehtiniemi et al and Bao et al suggest that NMD is critical for several steps of spermatogenesis, including the ProSG-spermatogonia transition, initiation of meiosis, and haploid germ cell progression (Fig. 2B). All these studies highlight the important role of NMD in promoting germ cell survival.

A recent study—Tan et al (Tan et al, 2024)—delved more deeply into the mechanism by which NMD impacts spermatogenesis. These authors chose to focus on SMG5, as they found that this NMD factor undergoes a translocation from the cytoplasm to the nucleus at the late-pachytene spermatocyte stage. This raised the possibility that SMG5 is critical at this stage, but the authors found that, instead, loss of SMG5 causes an earlier defect. They conditionally knocked out *Smg5* in ProSG (by breeding *Smg5*-floxed mice with *Ddx4*-Cre mice) and found that this causes a severe meiosis-entry defect (at P10). These *Smg5-*cKO mice have significantly reduced spermatogonial numbers (at P14), increased germ-cell apoptosis, and eventually a “Sertoli cell-only” phenotype. By analyzing spermatogonia dynamics before overt germ cell death (at P7 and P10), the authors found that SMG5 loss does not significantly disrupt spermatogonial proliferation, but instead is critical for their differentiation, as revealed by reduced numbers of differentiating (KIT^+^) spermatogonia. Transcriptomic analysis of P10 whole testes revealed that SMG5 deficiency causes NMD inhibition (based on the upregulation of several known NMD-target mRNAs), thereby supporting the notion that the spermatogonial defects observed by the authors are due to NMD deficiency. This was an important observation given that in other biological contexts, SMG5 knockdown has been reported to have only minor effects due to its being partially redundant with another NMD factor - SMG7 (Boehm et al, 2021; Huth et al, 2022; Metze et al, 2013). Tan et al also found that many genes known to be essential for spermatogonial expansion and differentiation are  downregulated in response to the loss of SMG5, while genes associated with the p38 MAPK signaling pathway were upregulated. Together, these findings suggest an essential role of NMD in regulating spermatogenic cell fate, germ cell signaling, and, ultimately, male fertility.

Putative NMD-target mRNAs have been identified in germ cells by several of the studies described above (Bao et al, 2016; Fanourgakis et al, 2016; Lehtiniemi et al, 2022; Tan et al, 2024). These putative NMD targets are candidates to act downstream of NMD to impact germ cell events. However, identifying bona fide NMD-target mRNAs in germ cells is challenging, given that disruption of NMD often leads to germ cell apoptosis, thereby confounding the interpretation of transcriptomic changes (Han et al, 2018; Hwang and Maquat, 2011). Thus, it is critical that time points prior to apoptosis are selected for transcriptomic analysis. In addition, it is important to independently define NMD-target mRNAs in the different germ cell stages and somatic cell types in the testis, particularly given the recent finding that NMD targets are often cell-type specific (Tan et al, 2025).

NMD is triggered by different signals. The most consistent signal that elicits NMD is an exon–exon junction ~55 nt or more downstream of the stop codon (dEJ) defining the main ORF (Box 1). Indeed, databases defining alternatively spliced mRNA isoforms that are degraded by NMD rely entirely on this robust NMD-inducing dEJ signal (Britto-Borges et al, 2024). Consistent with this, dEJ-containing transcripts have been found to be enriched among the upregulated transcripts after NMD is suppressed (Chousal et al, 2022; Hurt et al, 2013; Karousis et al, 2021; Tan et al, 2025). Surprisingly, however, Bao et al found that dEJ-containing transcripts were *not* enriched among transcripts upregulated in NMD-deficient (*Upf2*-cKO) testes or purified germ cell subsets relative to control testes and germ cells, respectively (Bao et al, 2016). Similarly, Lehtiniemi et al and Fanourgakis et al found that dEJ-containing transcripts were not enriched in the upregulated transcripts in round spermatids made NMD-deficient as a result of conditional loss of the NMD protein, SMG6, or the NMD-associated protein, TDRD6, respectively (Fanourgakis et al, 2016; Lehtiniemi et al, 2022). Together, these findings suggest the possibility that male germ cells are unique in *not* recognizing the dEJ NMD-inducing signal.

Another signal known to induce NMD is a long 3’UTR (Box 1) (Kishor et al, 2019; Kurosaki et al, 2019; Munoz et al, 2023). All three of the above studies found that long 3’ UTRs were enriched in candidate NMD-target mRNAs (transcripts upregulated in response to NMD deficiency) in germ cells (Bao et al, 2016; Fanourgakis et al, 2016; Lehtiniemi et al, 2022). Thus, it is likely that this NMD-inducing signal plays a major role in defining NMD-target mRNAs in some stages of germ cells. However, because no specific length of 3’UTR triggers NMD, it remains for future studies to elucidate which specific mRNAs harboring long 3’UTRs are degraded by NMD in germ cells.

It also remains for the future to determine whether germ cells are completely incapable of recognizing the dEJ NMD-inducing signal, and, if so, whether this escape from NMD is confined to specific germ cell stages. Knowing whether or not a dEJ can trigger NMD in germ cell stages is critical toward defining the full repertoire of NMD targets in these cell subsets, and thus understanding the regulatory mechanisms by which NMD acts to influence spermatogenesis.

The enigmatic NMD factor, UPF3A, has also been shown to be important for spermatogenesis. UPF3A is abundant in the testis, particularly in spermatocytes, which largely lack expression of its paralog—UPF3B (Shum et al, 2016). To test UPF3A’s function in spermatocytes, Shum et al conditionally knocked out UPF3A in germ cells just prior to the initiation of meiosis (by breeding *Upf3a-*floxed mice with *Stra8*-Cre mice), and found these UPF3A-deficient mice have a ~ten-fold reduction in sperm count relative to wild-type littermates (Shum et al, 2016). Histological analysis suggested that these *Upf3a*-mutant mice have a defect in spermatocyte progression.

The likely reason that spermatocytes uniquely depend on *Upf3a* is because its paralog, *Upf3b*, is on the X chromosome, and, thus, is transcriptionally shut down by meiotic sex chromosome inactivation, a mechanism that transcriptionally silences most X chromosome genes during meiosis (Turner, 2007). Hence, while *Upf3a* and *Upf3b* are well-established to act redundantly in a variety of cell types (Chan et al, 2007; Lykke-Andersen et al, 2000; Shum et al, 2016; Wallmeroth et al, 2022; Yi et al, 2022), this is not possible in spermatocytes and hence this germ-cell stage is likely to be entirely dependent on UPF3A (not UPF3B) for NMD.

NMD also appears to be critical for Sertoli cells, the giant nurse cells in contact with all stages of germ cells in the testis. This was shown by Bao et al, who found that conditional ablation of *Upf2* in Sertoli cells (via breeding with *Amh*-Cre mice), leads to severe testicular atrophy and male sterility in mice (Bao et al, 2015). RNAseq analysis of total testes from these cKO mice (at the perinatal (P4) stage) indicated that loss of NMD in Sertoli cells causes the accumulation of dEJ+ transcripts and dysregulates genes known to be critical for Sertoli cell development, such as *Wt1* and *Dmrt1*. It remains to be established which of these genes encodes direct NMD-target mRNAs in Sertoli cells and how their regulation by NMD is physiologically important for spermatogenesis.

### Other biological processes

NMD has also been implicated in other developmental processes. For example, Weischenfeldt et al showed that hematopoietic cell-specific deletion of *Upf2* (by breeding with *Mx1*-Cre mice) leads to the complete and lasting cell-autonomous extinction of all hematopoietic stem and progenitor populations (Weischenfeldt et al, 2008). Deletion of *Upf2* specifically in the T-cell lineage (by breeding with *Lck*-Cre mice) results in thymic atrophy, with significant reductions in thymocytes, accompanied by increased numbers of AnnexinV^+^ pre-apoptotic thymocytes. A later study—Frischmeyer-Guerrerio et al—found that ubiquitous expression of a dominant-negative form of UPF1 (dnUPF1) in mice results in perturbed fetal thymocyte development (Frischmeyer-Guerrerio et al, 2011). A key step in thymocyte development is the programmed rearrangement of T-cell receptor (TCR) genes necessary to generate the large repertoire of cell-surface TCR proteins essential to recognize diverse foreign antigens. Two-thirds of the time, these rearranged TCR genes are out-of-frame and thus have PTCs and give rise to mRNAs rapidly degraded by NMD (Carter et al, 1995; Gudikote and Wilkinson, 2002). Therefore, a possible explanation for the disrupted thymic development of dnUPF1 mice is an accumulation of PTC-bearing TCR mRNAs due to NMD disruption. Frischmeyer-Guerrerio et al obtained several lines of evidence in support of this possibility, including the finding that introduction of a functionally rearranged allele of the TCR-β gene rescued the thymic development defect in dnUPF1 mice.

In contrast to the negative effects of NMD disruption on proliferating hematopoietic cells, Weischenfeldt et al found that bone marrow-derived macrophages—which are post-mitotic—remain viable after loss of UPF2. This raises the possibility that NMD is mainly essential for proliferating cells—at least in the hematopoietic lineage (Weischenfeldt et al, 2008). To define candidate NMD-target mRNAs in hematopoietic cells, Weischenfeldt et al identified mRNAs upregulated in UPF2-deficient macrophages and thymocytes. These putative in vivo NMD targets included mRNAs encoding proteins involved in endoplasmic reticulum stress, protein degradation, and the unfolded protein response. Other upregulated mRNAs included alternatively spliced transcripts, mRNAs derived from expressed processed pseudogenes, and non-coding mRNAs. Several of these in vivo-defined putative NMD-target mRNAs, including *Snord22, Gas5*, and *Atf4* mRNA, have since been verified to be NMD-target mRNAs and are widely used to measure NMD deficiency.

NMD has also been implicated as playing key roles in the liver. As described above, loss of NMD specifically in developing liver endodermal cells leads to perinatal lethality due to failure of terminal differentiation (Thoren et al, 2010). To address the role of NMD in the adult liver, *Upf2* was inducibly knocked out in the adult quiescent liver cells (by breeding with inducible *Mx1*-Cre mice). This resulted in “fatty liver disease” (hepatosteatosis) and disruption of liver homeostasis (Thoren et al, 2010). These NMD-deficient cKO mice became moribund 3 weeks after pIC-induced *Upf2* KO. Livers from these NMD-deficient cKO mice also failed to regenerate following partial hepatectomy.

The circadian rhythm (or cycle) is a natural process influencing a wide variety of responses that repeats approximately every 24 h. To determine whether NMD influences this oscillatory process, Katsioudi et al examined the effect of mutations in the *Smg6* NMD gene on a circadian reporter (Katsioudi et al, 2023). They found that knock-in of a *Smg6* mutant that selectively lacks the NMD function of SMG6 caused a strong period-lengthening phenotype (Katsioudi et al, 2023). Because this *Smg6* mutant was still capable of mediating SMG6’s telomere and genome stability functions (Azzalin and Lingner, 2006), this provided evidence that SMG6’s NMD function is specifically required for the circadian rhythm. The authors also discovered that CRY2—a key transcriptional repressor within the circadian rhythm-generating feedback loop during the dark phase—is encoded by a NMD-target mRNA. This raises the possibility that this target has a role in NMD’s function in circadian rhythm.

### Approaches to study NMD’s in vivo functions and develop NMD modulatory therapy

**Figure 3:**
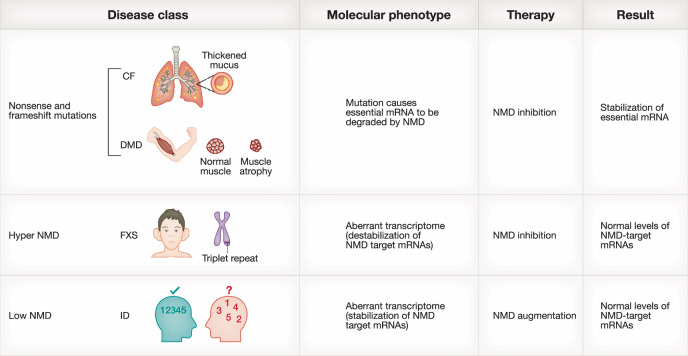
NMD-modulatory therapy strategies to treat disease. Nonsense and frameshift mutations have two effects: (i) they lead to generation of a mutant truncated protein and (ii) they typically trigger NMD, thereby destabilizing the mRNA encoding the truncated protein. In cases in which the truncated protein is still functional, NMD is counter-productive. NMD inhibitory therapy reverses this negative effect of NMD. Examples of diseases that are candidates to be treated with this approach include cystic fibrosis (CF) and Duchenne muscular dystrophy (DMD) (Amar-Schwartz et al., 2023; Keenan et al., 2019). In cases in which the truncated protein is not functional, NMD inhibitory therapy coupled with nonsense codon read-through therapy allows for production of full-length protein. For diseases that aberrantly increase NMD activity, such as Fragile X syndrome (FXS) (Kurosaki et al., 2021b), NMD inhibitory therapy would reverse this hyper-NMD phenotype and thereby potentially rescue the stability of key mRNAs that have a role in the intellectual disability (ID) and other defects associated with FXS. Conversely, for diseases associated with reduced NMD activity—such as ID and neurodevelopmental disorders caused by mutations in NMD factor genes such as UPF3B—NMD-enhancing therapy would be predicted to rescue such defects.

To better understand the roles of NMD during development in vivo, it would be beneficial to have a mouse model that transiently suppresses NMD at desired developmental time points. In addition, such a mouse model could be used to assess potential future drug therapies that modulate NMD as a treatment for human diseases (Fig. 3). As a potential example of the latter, Duchenne muscular dystrophy (DMD) is often caused by nonsense mutations in the dystrophin gene (Fig. 3). By suppressing the NMD-induced decay of such nonsense-mutant transcripts, NMD inhibition can increase the translation of truncated but still functional forms of the dystrophin protein (Amar-Schwartz et al, 2023). Likewise, the ~10% of cystic fibrosis patients harboring nonsense mutations in the *CFTR* gene could potentially benefit from NMD suppression therapy (Keenan et al, 2019) (Fig. 3). NMD modulatory therapy may also have a role in treating cancer; e.g., by upregulating the expression of frameshift-derived immunogenic peptides from tumors with defective DNA mismatch repair (Becker et al, 2021; Tan et al, 2022). More detailed information on future therapeutic NMD approaches can be found in a recent review (McMahon and Maquat, 2025).

To develop a NMD suppression therapy, a key concern is whether global NMD attenuation is safe, particularly in light of the many studies, described above, providing evidence that NMD has roles in normal biological processes. To evaluate this, Echols et al generated a transgenic mouse model that expresses an inducible, dominant-negative form of human UPF1 as a means to inhibit endogenous NMD activity in mouse tissues (Echols et al, 2020). Treatment of these dnUPF1-transgenic mice with a transcriptional inducer of the dnUPF1 transgene, doxycycline (Dox), caused modest-to-strong NMD inhibition, depending on the tissue examined. These authors found no discernable defects when dnUPF1 expression was induced to mild-to-moderate levels, indicating that modest global NMD attenuation is generally well tolerated. Importantly, modest NMD inhibition was also found to significantly enhance in vivo PTC suppression in a test disease gene, suggesting clinical efficacy. Of note, however, mice induced to express high levels of dnUPF1 had significantly increased numbers of several leukocyte subsets, suggesting an inflammatory response. In addition, statistically significant bone alterations were observed in female mice (but not male mice) induced to express high levels of dnUPF1.

Another approach that has been used to inhibit NMD in vivo is antisense oligonucleotides (ASOs) targeting NMD factors (Huang et al, 2018a). Subcutaneous introduction of adult mice with low doses of ASOs targeting *Upf1, Up2, Upf3b, Smg1*, and *Smg6* decreased the expression of the corresponding NMD factor in the liver with only minimal toxicity. Together, this ASO approach and dnUPF1-transgenic mice can be used for pre-clinical studies to test NMD suppression therapy, as well as to study the mechanisms by which NMD acts in biological processes.

## Stem cell models as avenues to understand NMD’s developmental roles

Stem cell models have been developed as powerful tools to study specific developmental events. In this section, we focus on studies that have used stem cells to study the roles of NMD in developmental processes.

### Embryonic stem cells (ESCs)

As described above, loss of any of several different NMD factor genes causes early embryonic developmental defects in mice, strongly suggesting that NMD is critical for early embryonic development (Chousal et al, 2022; Kurosaki et al, 2019; Li et al, 2015; McIlwain et al, 2010; Medghalchi et al, 2001; Shaheen et al, 2016; Shum et al, 2016; Weischenfeldt et al, 2008). Since ESCs model some of the events that occur during early embryonic development (Murry and Keller, 2008), investigators have used ESCs to investigate specific roles of NMD in embryonic development, particularly during pre-implantation development. Early attempts to achieve this failed because initial studies found that ESCs could not be derived from NMD-deficient mice. For example, Medghalchi et al found *Upf1*-KO ICM cells underwent apoptosis after 72 h in culture, preventing ESC derivation (Medghalchi et al, 2001). Likewise, Li et al found that *Smg6*-KO ESCs could not be generated from *Smg6*-null mice because *Smg6*-KO ICM cells failed to grow in cultures after 5 days (Li et al, 2015). To overcome this, Li et al used a “Trojan horse” approach in which they mated *Smg6-*floxed mice with mice expressing an inducible form of CRE (CreERT2). This allowed the authors to isolate “NMD-active” ESCs that only lose *Smg6* after CRE expression is induced at the ESC stage. Another study—Chousal et al (Chousal et al, 2022)—defined another approach to generate NMD-deficient ESCs. These authors discovered that while *Upf2*-KO ESCs could also not be generated under primed conditions (akin to the post-implantation epiblast state), they *could* be generated under naive culture conditions (akin to the pre-implantation epiblast state). This finding was not only of practical significance, but it raised the possibility that NMD is required for “primed state survival” and/or the transition from the naive to the primed state.

The ability of Li et al to generate NMD-deficient ESCs (Li et al, 2015) allowed them to address questions about the role of NMD during early development. First, they asked whether NMD has a role in the maintenance of undifferentiated ESCs grown under primed conditions. They found that *Smg6*-KO ESCs grown under these conditions proliferate normally and are morphologically indistinguishable from control ESCs (Li et al, 2015). Cell cycle and apoptosis analyses confirmed that SMG6 is dispensable for ESC viability and self-renewal. Next, the authors asked whether NMD has a role in the differentiation of ESCs. They found that *Smg6*-KO ESCs fail to differentiate both in vitro and in vivo. In vitro, *Smg6*-KO ESCs failed to downregulate pluripotency markers under conditions that normally promote embryoid body formation (removal of LIF) or lineage-specific differentiation events (addition of retinoic acid or DMSO). In vivo chimera analysis showed these cells could not differentiate into any of the three germ layers.

While these data clearly showed that SMG6 is critical for ES cell differentiation, they did not prove a role for NMD in this event, as SMG6 has known non-NMD roles. To distinguish between the NMD and non-NMD roles of SMG6, Li et al performed ‘rescue experiments’ with mutant forms of SMG6. They found that mutants lacking telomerase-promoting activity, but not those lacking NMD-promoting activity, rescued ES cell differentiation. This provided evidence that the NMD activity of SMG6 is responsible for driving ESC differentiation. As further evidence, the authors found that depletion of several NMD factors in addition to SMG6 (UPF1, UPF2, SMG1, and SMG5) also caused a defect in ESC differentiation. Together, these findings strongly suggest that NMD is essential for mouse ESC differentiation.

Li et al also investigated the underlying mechanisms by which NMD drives ESC differentiation (Li et al, 2015). Through RNAseq analysis of *Smg6*-KO ESCs, they found that pluripotency genes exhibit sustained expression after SMG6 loss. Among them was *c-myc*, which they regarded as a particularly attractive candidate to be a NMD effector for several reasons. First, C-MYC is a major pluripotency factor with well-defined actions in a wide variety of cellular contexts (Chappell and Dalton, 2013). Second, the authors obtained evidence that *c-myc* mRNA is a NMD-target mRNA. Third, they observed that *c-myc* mRNA was specifically upregulated by SMG6 mutants harboring an intact NMD-promoting domain. Finally, the authors discovered that many SMG6-regulated mRNAs had previously been shown to also be C-MYC-regulated genes. To directly assess whether C-MYC has a functional role downstream of SMG6, the authors performed both mimic and rescue experiments. They found that overexpressing C-MYC in normal ESCs inhibited their ability to differentiate, thereby mimicking the differentiation defect in *Smg6*-KO cells, which express elevated levels of C-MYC. Reversing the abnormally high expression of C-MYC in *Smg6*-KO ESCs (using RNAi) caused these cells to acquire the ability to differentiate. Together, these data supported the existence of a NMD-based molecular circuit involving *c-myc* that is critical for ESC differentiation.

A recent study—Huth et al (Huth et al, 2022)—provided further insights into how the NMD pathway regulates ESC differentiation. They obtained several lines of evidence that NMD susceptibility is hardwired into the translation initiation factor gene, *eIF4a2*, in a unique fashion that influences ESC identity and fate. These authors found that a conserved PTC-containing *eIF4a2* mRNA isoform—which they called “*Eif4a2*^*PTC*^”—is a NMD-target mRNA whose upregulation in NMD-deficient ESC causes their differentiation delay. Through RIPseq and co-IP/MS analyses, the authors found that NMD deficiency in ESCs leads to translation of the truncated EIF4A2^PTC^ protein, which elicits increased mTORC1 activity coupled with an increased translation rate and a consequent differentiation delay. Given that NMD itself is translation-dependent (Belgrader et al, 1993; Carter et al, 1995), these data indicate the existence of an intriguing feedback loop between NMD and the translation machinery that influences differentiation and cell identity. To draw their conclusions, Huth et al knocked out the *Smg5*, *Smg6*, and *Smg7* genes, either singly or in combination. Their experiments revealed a clear hierarchy in the severity of NMD target deregulation and differentiation phenotypes between these SMG factor KOs: *Smg5-*KO > *Smg6-*KO > *Smg7-*KO. The authors also found that some SMG factors act synergistically to promote exit from pluripotency in a manner consistent with their known roles in UPF1 dephosphorylation, SMG factor recruitment to UPF1, and RNA decay.

Interestingly, NMD appears to function differently in human ESCs than in mouse ESCs. Lou et al knocked down UPF1 and UPF3B in human ESCs and found that while this inhibited their differentiation into mesoderm, it *promoted* their differentiation into definitive endoderm (Lou et al, 2016). This yin-yang result suggested that NMD acts as a molecular switch influencing lineage specification. In support of this “switch model,” Lou et al found that NMD magnitude is differentially regulated in human ESCs as they differentiate into the three germ layers. This NMD switch may be controlled through differential expression of NMD factors, as Lou et al found that NMD factors are differentially regulated during the  differentiation of the 3 germ layers. Regardless of the precise mechanism, the finding that mesoderm and endoderm formation are oppositely influenced by NMD is a striking example of NMD influencing cell fate.

Recently, Jung et al obtained evidence that NMD also influences the formation of the third germ layer—ectoderm (Jung et al, 2024). This discovery came as a result of the authors’ finding that the RNA-binding protein, LIN28A, competes with the NMD factor, UPF2, for binding to the NMD factor UPF1. These authors found that this interaction is functionally important, as they found that NMD-target RNAs levels are raised by LIN28A overexpression, suggesting that LIN28A suppresses NMD. These authors then went on to show that this UPF1-LIN28A interaction is also physiologically important, as it maintains pluripotency and specifically inhibits differentiation to the ectoderm lineage, not the mesoderm or endoderm lineage. Together with validation of these findings through immunostaining of ectodermal markers, this study suggested a role for NMD in making self-renewal vs. ectoderm fate decisions.

Together, these human and mouse ESC studies reveal nuanced and stage-specific roles for NMD in ESC fate decisions. In human ESCs, NMD has differential effects on the differentiation of pluripotent cells into the 3 germ layers. In contrast, in mouse ESCs, NMD broadly promotes ESC differentiation (Li et al, 2015) and influences its timing (Huth et al, 2022), consistent with NMD’s in vivo role of promoting epiblast progression (Chousal et al, 2022). It remains for future studies to use ESCs to elucidate NMD’s early developmental roles in more depth (e.g., by comparing naive vs. primed ESCs) and to address several burning questions, including determining which of NMD’s roles are conserved vs. species-specific.

By what molecular mechanisms does NMD influence ESC differentiation? Lou et al identified numerous putative NMD-target transcripts in human ESCs that encode components of signaling networks and regulators of pluripotency and differentiation, including TGFβ/BMP, WNT, and FGF signaling factors (Lou et al, 2016). This led the authors to directly test the role of TGFβ/BMP signaling in NMD action in human ESCs. Through UPF1 and UPF3B knockdown experiments, the authors obtained evidence that NMD influences the conversion of human ESCs into endoderm vs. mesoderm via regulation of TGFβ/BMP signaling.

A recent study provided evidence that the NMD-target mRNAs in human ESCs are relatively unique. Using genome-wide approaches coupled with stringent criteria to define high-confidence NMD-target RNAs in human ESCs, Tan et al discovered that there is very little overlap between these NMD targets and those in NPCs (Tan et al, 2025). Even when considering only transcripts co-expressed between the two cell types, most NMD targets were either ESC- or NPC-specific. The discovery that NMD-target RNAs can be largely cell-type specific implies that the in cis signals that trigger NMD (e.g., dEJs and long 3’UTRs) are not always sufficient to elicit NMD and that cellular context may be a major determinant of whether or not an mRNA is degraded by the NMD pathway. This, in turn, underscores the need to empirically define the profile of NMD-target RNAs in every biological context one is interested in studying.

The human and mouse ESC studies that have been performed to date provide clear evidence that NMD is critical for pluripotency and ESC fate. However, the precise roles of NMD appear to depend on the species of origin and the particular cell lineage being investigated. These roles in different contexts require being investigated in depth. Another key question for the future is whether the NMD-driven mechanisms identified in ESCs apply to in vivo embryonic development.

### Neural stem and progenitor cells

The role of NMD has also been studied in neural stem and progenitor cells. For example, Lou et al found that the NMD efficiency is reduced during in vitro neural differentiation of both mouse neural stem cells (NSCs) and human NPCs (Lou et al, 2014). As evidence for causality, force-expressing NMD factors to prevent NMD downregulation inhibited neural differentiation. To test whether NMD downregulation is sufficient for neural differentiation, the authors depleted UPF1 via RNAi and found that this was sufficient to trigger the differentiation of mouse NSCs derived from fetal (E14.5) brain tissue. NMD-target mRNAs encoding pro-neural proteins were enriched in undifferentiated pre-neural cells, raising the possibility that NMD promotes the undifferentiated cell state by degrading mRNAs encoding proteins that drive neural differentiation. This was empirically supported by mimic and rescue experiments demonstrating that a NMD target encoding the key pro-neural differentiation factor—SMAD7—must be degraded by NMD to maintain pre-neural cells and NSCs in an undifferentiated state. Finally, Lou et al identified several miRNAs targeting NMD factors that control NMD activity. These miRNAs were found to act in a bistable feedback circuit that stabilizes the NSC state or promotes neural differentiation when NMD magnitude is high or low, respectively.

While the data from Lou et al (Lou et al, 2014), above, indicated that the NMD pathway, as a whole, maintains the neural stem cell state and thus *prevents* neural differentiation, there is also evidence that the specific NMD factor, UPF3B, can *promote* neural differentiation. Jolly et al found that depletion of UPF3B (by RNAi) in mouse NPCs (derived from E18.5 frontal cortex) reduced their ability to differentiate. As further support, another study—Huang et al—found that NSCs isolated from *Upf3b*-KO mice exhibit hyper-self-renewal and poorly differentiate when cultured under conditions that efficiently differentiate control mouse NSCs (Huang et al, 2018b). One possible explanation for UPF3B acting differently than the NMD pathway as a whole is the recent evidence that UPF3B promotes the decay of a largely different set of NMD-target RNAs than the broadly acting NMD factor UPF2 (Tan et al, 2025).

Using a new computational pipeline, factR2, Zhuravskaya et al recently reported evidence that NMD collaborates with alternative splicing to regulate the development of neurons from pluripotent stem cells (Zhuravskaya et al, 2024). These authors generated an inducible neuronal differentiation system involving a mouse *Ngn2* transgene knocked into an ESC line under the control of a Dox-inducible promoter. Using this single-step neural differentiation system coupled with factR2, they identified hundreds of alternatively spliced transcripts that contain a dEJ and thus are candidates to be targeted for decay by NMD (“AS-NMD” transcripts) at different time points as ESC cells transition to begin to form neurons. They observed that genes downregulated during neuronal differentiation exhibited a striking increase in the frequency of such AS-NMD transcripts. This provided support for the possibility that NMD coupled with alternative splicing is critical for the downregulation of non-neuronal genes during the differentiation of neurons from stem cells.

## Perspective

We suggest the following are three important future topics of investigation: (i) deeper and more broad investigation into the roles of NMD in different biological scenarios, including scrutiny as to which of NMD’s biological roles are fundamental vs. secondary; (ii) assessment as to whether NMD has critical roles *after* embryonic and postnatal development, as the answer to this question will determine whether NMD modulator therapy is feasible for therapeutic purposes in adults; and (iii) elucidating the underlying molecular mechanisms by which NMD acts to influence biological events.

### What are the fundamental biological roles of NMD?

In this review, we have discussed various biological scenarios in which NMD is proposed to function. However, it remains to be determined what NMD specifically does in these different scenarios. For example, with few exceptions, the underlying cellular mechanisms responsible for the developmental defects identified in NMD-deficient organisms have not been determined. Are the developmental defects observed due to perturbed differentiation, altered cell proliferation, reduced cell survival, or some combination of these events? In most cases, this has not been addressed. Even in cases in which this question has been partially addressed (Chousal et al, 2022; Huth et al, 2022), the role of NMD in determining the fate of each of the cell populations involved remains to be scrutinized; e.g., by lineage tracing or single-cell RNAseq analyses.

A particularly vexing concern that remains unexplored is whether the phenotypes observed when NMD factors are knocked down or knocked out are merely because of a secondary role of NMD. For example, if NMD perturbation decreases cell proliferation or triggers apoptosis, as has often reported to be the case (Avery et al, 2011; Bao et al, 2016; Chousal et al, 2022; Lin et al, 2024; Lou et al, 2014; Medghalchi et al, 2001; Nelson et al, 2016; Tan et al, 2024; Weischenfeldt et al, 2008), there is a legitimate concern that this is simply because NMD deficiency causes the cell type in question to become “sick.” For example, the transcriptome misregulation caused by NMD perturbation might merely elicit broad metabolic defects that secondarily lead to reduced cell proliferation or increased cell apoptosis.

How can one determine if NMD-deficiency causes a primary defect versus a secondary defect? One approach is to ask whether the gain-of-function phenotype is compatible with  the loss-of-function phenotype. For example, to determine whether NMD has a primary role in promoting cell proliferation, one can ask not only whether inhibited NMD reduces cell proliferation, but also whether elevated NMD *increases* cell proliferation. If NMD’s only role in the proliferative response is secondary (e.g., to maintain “cell health”), it is unlikely that elevating NMD would increase cell proliferation. Lou et al used such a gain- and loss-of-function approach to address this question in neural precursor cells and found that UPF1 knockdown and overexpression decrease and increase their proliferation, respectively, providing evidence that NMD acts in a primary manner in this particular cellular type (Lou et al, 2014). However, this might not be the case for all other cell types. Another approach to address primary vs. secondary effects is to determine whether there are NMD-target mRNAs that conform to the hypothesis that NMD is directly involved in the biological event in question. For example, if NMD has a fundamental role in promoting cell proliferation of a given cell type, this predicts that anti-proliferation factors are encoded by NMD targets in that cell type (Lin et al, 2024; Lou et al, 2014).

Another important question is whether NMD has conserved roles. At present, it is not clear if even relatively related organisms (rats and mice or humans and non-human primates) use NMD for a common purpose. Discovering conserved biological roles for NMD will support its physiological significance. By analogy, the HOX homeobox transcription factors, which were originally discovered in flies, have since been shown to be regulated in a similar manner and have related developmental functions across the phylogenetic scale (Pick and Au, 2025).

### Will NMD modulatory therapy be feasible?

There is considerable interest in modulating NMD as a means to treat diseases caused by nonsense mutations in disease genes (Martins-Dias and Romao, 2021) or mutations that activate or suppress NMD (Kurosaki et al, 2021a; Kurosaki et al, 2021b; Tarpey et al, 2007). However, it is currently unknown whether NMD modulation therapy will cause unacceptable side effects. Because NMD has been suggested to be involved in a wide variety of functions (as summarized in this review), a reasonable view is that NMD modulatory therapy will likely elicit unacceptable side effects. However, if NMD’s primary roles are confined to embryonic development, NMD modulatory therapy may instead be well tolerated. Thus, a critical goal of future investigations should be to address which of these opposing views is correct. The answer may be nuanced. For example, NMD may have subtle roles after birth whose perturbation by NMD therapy may lead to some side effects, but these are tolerable. Or NMD may have important roles during child development that prevent NMD modulatory therapy for children, but allow for NMD modulatory therapy in adults.

To address these questions, it is critical to first perform in-depth systematic studies on NMD’s roles in experimental animal models of different ages. In this review, we highlighted a mouse model generated by Echols et al that allows NMD to be transiently and precisely perturbed during the time window of interest (Echols et al, 2020). It will be important to also develop other experimental models to address the relative importance of NMD’s different hypothesized biological roles in vivo.

NMD appears not to be a single linear pathway. Instead, evidence suggests that NMD has different “branches” that depend on different NMD factors and regulate different sets of NMD-target mRNAs (Yi et al, 2021). This has important therapeutic implications, as it means that manipulation of only a single branch of NMD is likely to cause less side effects than manipulation of the entire NMD pathway. Even if future research reveals that discrete NMD branches with fundamentally different roles do not exist, it is clear that perturbation of different NMD factors leads to upregulation of different groups of NMD targets (Asthana et al, 2022; Colombo et al, 2017; Ge et al, 2016; Huang et al, 2011; Huang et al, 2018b; Hurt et al, 2013; Huth et al, 2022; Imamachi et al, 2017; Mabin et al, 2018; Nelson et al, 2016; Tan et al, 2025; Yi et al, 2021). This, in turn, raises the possibility that therapeutic perturbation of some NMD factors can achieve a therapeutic goal with little or no toxicity. In support, Huang et al examined the effect of ASO-mediated knockdown of different NMD factors in mice and found that systemic knockdown of the NMD “branch” factor, UPF3B, increased expression of PTC-bearing disease genes with no obvious toxicity (Huang et al, 2018a). As further evidence for UPF3B being unique in its specificity, a recent study showed that UPF3B promotes the decay of a set of NMD-target mRNAs that only partially overlaps with those targeted for decay by the core NMD factor, UPF2, in both human ESCs and NPCs (Tan et al, 2025). To further explore this concept, more investigations examining the effects of manipulating different NMD factors in different disease settings are critical.

Another issue that needs to be addressed in more depth is the “*non*-NMD” functions of NMD factors. As described in this review, some NMD factors have roles in DNA damage responses and telomere integrity maintenance (Han et al, 2018; Hwang and Maquat, 2011), and UPF1 has been shown to function in many RNA decay pathways in addition to NMD (Lavysh and Neu-Yilik, 2020). These non-NMD functions complicate strategies to manipulate NMD factors for therapeutic benefit. Using *SMG6* mutants that differentially perturb NMD vs. SMG6’s other functions, there has been progress in determining which of SMG6’s biological functions actually depend on NMD (Katsioudi et al, 2023; Li et al, 2015). This genetic dissection approach needs to be extended to other NMD factors.

### How does NMD achieve its functions at the molecular level?

There is considerable evidence that NMD regulates biological events by degrading specific mRNAs encoding proteins involved in that biological event (Colak et al, 2013; Huth et al, 2022; Katsioudi et al, 2023; Li et al, 2015; Lin et al, 2024; Lou et al, 2014; Nelson et al, 2016; Notaras et al, 2020). It is important to expand such efforts, not only because this will provide basic knowledge about how NMD functions in different biological contexts, but because this may, in turn, help define the paths towards treating diseases with NMD modulatory therapy.

While there has been some progress in ascertaining the underlying molecular mechanisms by which NMD influences biological events in cultured cells, virtually nothing is known about this topic in vivo. In part, this stems from the technical challenges of performing the “mimic” and “rescue” experiments necessary to define which of the sea of NMD-target mRNAs have a causal role downstream of NMD in a given biological scenario in vivo. To make such in vivo experiments more feasible, it will be important to narrow down candidate functional NMD targets by doing mimic and rescue experiments in cultured cells.

NMD is a highly regulated pathway (Aznarez et al, 2018; Huang and Wilkinson, 2012; Karam et al, 2013). This regulation is important, as it is a logical means by which NMD can influence biological events. By regulating the magnitude of NMD, the scores of NMD-target mRNAs in a given biological setting will, in turn, be stabilized or more rapidly degraded (when NMD is down- or upregulated, respectively), leading to changes in the levels of their encoded proteins. One can easily imagine that such a global shift in NMD-target mRNA levels will be physiologically important, e.g., for developmental transitions. To quantify the NMD magnitude changes that mediate these global changes in NMD-target RNA expression, many NMD reporters have been generated (Alexandrov et al, 2017; Boelz et al, 2006; Kolakada et al, 2024; Paillusson et al, 2005; Sato and Singer, 2021). These reporters, as well as other approaches, have been used to define changes in NMD magnitude in cells as they undergo transitions in vitro (Chousal et al, 2022; Lou et al, 2016; Pereverzev et al, 2015; Sato and Singer, 2021). A critical future goal is to develop NMD reporters that monitor NMD magnitude changes in vivo. This will open the door to elucidating how NMD-based switches influence biological events in their normal setting. Ultimately, this knowledge has the potential to pave the way for novel therapeutic strategies targeting the NMD pathway to treat disease.

## Supplementary information

Peer Review File
