# Author Correction: Beyond quality control: biological roles of nonsense-mediated RNA decay

**DOI:** 10.1038/s44318-026-00822-z

**Published:** 2026-06-02

**Authors:** Kun Tan, Miles F Wilkinson

**Affiliations:** 1 Department of Obstetrics, Gynecology, and Reproductive Sciences, University of California San Diego, La Jolla, CA USA; 2 Institute of Genomic Medicine, University of California San Diego, La Jolla, CA USA

**Correction to:** *The EMBO Journal*. 10.1038/s44318-026-00794-0 | Published online 09 May 2026

Figure placement for Figures 2 and 3 are corrected.

Figures 2 and 3 were placed incorrectly. The journal corrects this error.

