# Supplementary material for: Beyond quality control: biological roles of nonsense-mediated RNA decay
Source: EMBO J. 2026 May 9;45(13):4361–78. doi: 10.1038/s44318-026-00794-0 (PMC13323391; doi:10.1038/s44318-026-00794-0)
Supplement: Supplementary file 1 — Peer Review File [file 44318_2026_794_MOESM1_ESM.pdf]

THE EMBO JOURNAL

Beyond quality control: biological roles of nonsense-mediated RNA decay

Kun Tan and Miles Wilkinson

Corresponding author(s): Miles Wilkinson (mfwilkinson@health.ucsd.edu) , Kun Tan (kutan@health.ucsd.edu)

Review Timeline:

Submission Date:	15th Jul 25
Editorial Decision:	7th Sep 25
Revision Received:	17th Sep 25
Editorial Decision:	19th Jan 26
Revision Received:	20th Jan 26
Accepted:	10th Apr 26

Editor: Cornelius Schneider

Transaction Report:

(Note: With the exception of the correction of typographical or spelling errors that could be a source of ambiguity, letters and reports are not edited. Depending on transfer agreements, referee reports obtained elsewhere may or may not be included in this compilation. Referee reports are anonymous unless the Referee chooses to sign their reports.)

1st Editorial Decision 7th Sep 2025

Dear Prof. Wilkinson,

Thank you for submitting your review proposal for consideration by the EMBO Journal. It has now been seen by three referees whose comments are shown below.

As you can se from the reports all three referees think that the topic is interesting but also give several suggestions how to improve the manuscript further and I think that most of the suggestions are constructive and would improve the manuscript significantly.

Given the referees' positive recommendations, I would like to invite you to submit a revised version of the manuscript, addressing the comments of all three reviewers. I should add that it is EMBO Journal policy to allow only a single round of revision, and acceptance of your manuscript will therefore depend on the completeness of your responses in this revised version.

When preparing your letter of response to the referees' comments, please bear in mind that this will form part of the Review Process File, and will therefore be available online to the community. For more details on our Transparent Editorial Process, please visit our website: https://www.embopress.org/page/journal/14602075/authorguide#transparentprocess

We generally allow three months as standard revision time. As a matter of policy, competing manuscripts published during this period will not negatively impact on our assessment of the conceptual advance presented by your study. However, we request that you contact the editor as soon as possible upon publication of any related work, to discuss how to proceed. Should you foresee a problem in meeting this three-month deadline, please let us know in advance and we may be able to grant an extension.

Thank you for the opportunity to consider your work for publication. I look forward to your revision.

Yours sincerely,

Cornelius Schneider, PhD
Editor
The EMBO Journal
c.schneider@embojournal.org

Instructions for preparing your revised manuscript:

Please make sure you upload a letter of response to the referees' comments together with the revised manuscript.

Please also check that the title and abstract of the manuscript are brief, yet explicit, even to non-specialists.

When assembling figures, please refer to our figure preparation guideline in order to ensure proper formatting and readability in print as well as on screen:
https://bit.ly/EMBOPressFigurePreparationGuideline
See also guidelines for figure legends: https://www.embopress.org/page/journal/14602075/authorguide#figureformat

At EMBO Press we ask authors to provide source data for the main manuscript figures. You will receive a separate email with instructions for providing source data with your revised manuscript, including how to upload and organize the files.

IMPORTANT: When you send the revision we will require
- a point-by-point response to the referees' comments, with a detailed description of the changes made (as a word file).
- a word file of the manuscript text.
- individual production quality figure files (one file per figure)
- a complete author checklist, which you can download from our author guidelines (https://www.embopress.org/page/journal/14602075/authorguide).
- Expanded View files (replacing Supplementary Information)
Please see out instructions to authors
https://www.embopress.org/page/journal/14602075/authorguide#expandedview
- a Reagents and Tools Table as part of the Methods section, which can be downloaded from our author guidelines (https://www.embopress.org/page/journal/14602075/authorguide#structuredmethods)

Please remember: Digital image enhancement is acceptable practice, as long as it accurately represents the original data and conforms to community standards. If a figure has been subjected to significant electronic manipulation, this must be noted in the figure legend or in the 'Materials and Methods' section. The editors reserve the right to request original versions of figures and the original images that were used to assemble the figure.

Further information is available in our Guide For Authors: https://www.embopress.org/page/journal/14602075/authorguide

We realize that it is difficult to revise to a specific deadline. In the interest of protecting the conceptual advance provided by the work, we recommend a revision within 3 months (6th Dec 2025). Please discuss the revision progress ahead of this time with the editor if you require more time to complete the revisions. Use the link below to submit your revision:

Referee #1:

This review starts with a brief but sufficient overview of the basis of NMD, followed by a detailed review of work on mouse models and stem cell studies. The review reinforces the branched nature of NMD and reflects on its roles in embryonic development, neural development and signalling, different steps in spermatogenesis, hematopoietic cell proliferation, liver homeostasis and the circadian rhythm. The review ends with a consideration of future work to address the specific roles and molecular mechanisms of NMD, and the feasibility of NMD modulation therapy.
it is engagingly written and reflects an extensive review on the impact of NMD developmental and cell differentiation pathways in mammalian systems. I only have a few minor comments that are listed below.

There are few grammar errors:
Line 224 To define the underlying mechanism,...
Line 247 I don't think the abbreviation NPC (neural progenitor cell?) is defined
Line 235 The phrase OSN neurons is tautological, given OSN is the abbreviation of olfactory sensory neuron
Line 338 I don't think that the abbreviation MVH is explained.
Line 715 opposing views...
line 738 strange phrasing: maybe compared with instead of than ?
line 760 similar strange phrase: by which instead of how ?

There are cases where citations are embedded within hyphens (e.g. Notaras et al. line 284; Tan et al. line 356; Frishmeyer-Guerrerio et al. line 435; Chousal et al. line 523; Huth et al. line 562) (I may not have included all examples here), which seems unnecessary - why not simply have the embedded citation?

Some references appear to lack page numbers: lines 805, 849, 854, 867, 876, 947, 967, 1031, 1062, 1064, 1092

Fig. 1 perhaps emphasise this relates to roles of NMD in mammalian cells?

Fig 4 This is an effective figure but it would be clearer to me if it were rearranged to show, from left to right, the disease (e.g. CF), the condition (e.g. nonsense mutations), the NMD therapy (e.g. inhibtion) and the effect (e.g. increased expression of disease gene). The middle section labelled "Condition" is actually the consequence of the treatment rather than condition of the disease state, which is confusing.

Table 1 The column headed Non-NMD functions includes both functions of NMD factors (e.g. DNA damage response) and substrates (?) (E3 ligase and PI3K family member).

Referee #2:

General summary and opinion about the principal significance of the study, its questions and findings

Mechanistic details provided at the beginning of the paper detract from the strength of the paper. Many sentences there and throughout are misleading or incorrect. This review is on an important topic but requires re-writing for clarity, accuracy and the complete and balanced story (including references).

Specific major concerns essential to be addressed to support the conclusions

1. There are many examples of mechanistic details at the start of the paper that are misleading, incomplete or incorrect. Here is a partial list.
The first paragraph of the Introduction applies largely to yeast and should be clarified as such since what led to the "mini-field", as the authors call it, are the natural NMD targets in mammalian cells, which have no counterpart in yeast.
Third paragraph: Probably the first "normal" mRNA degraded by NMD that encodes a full-length protein was described much earlier (1998, on glutathione peroxidase mRNA) than those referenced. Also, the reference to LeLivelt and Culbertson is off-topic - on surveillance (see comment above).
Line 52. UPF2 and UPF3 are now considered auxiliary NMD factors, unlike UPF1. The author essentially writes this when calling UPF3B an "NMD branch" factor. In this context, though, what is UPF3A?
Line 65. How has this notion been challenged?
Lines 69-74. This is all true, but it does not do the EJC justice, omitting lots of important information. For example, while the "accessory protein" CASC3 is recruited to the EJC in the cytoplasmic, so is UPF2, which was previously defined as one of the three key NMD factors, but on a different EJC.
Line 87. How is this an exception to the "downstream exon-exon junction rule"?
Line 94. This paragraph is very confusing. First, branches are defined as NMD pathways that are either EJC-dependent or not, then uORFs are mentioned. It is unclear how the authors can consider branches as also defined by UPF3B, RNPS1 and CASC3, which returns to an EJC-dependent pathway, presumably. What about AKT?
2. Starting with line 106, there is insufficient information in the sentences to understand significance. E.g. what are "NMD-deficient Caenorhabditis elegans" and, considering the next sentence, do they have a shortened lifespan - how was NMD shown to increase lifespan?

Minor concerns that should be addressed (incomplete list)

Line 178. I wouldn't think that a putative repressor of NMD is "a non-NMD function".
Line 190. It has been shown that TA can also upregulates the nonsense-containing gene. Also, "paralogs of gene" should be "paralogs of some genes".
Line 211. Is it that NMD promotes cell proliferation or that the regulated inhibition of NMD promotes apoptosis?
Line 233. Please specify how the UFP3B targets were identified.
Line 241. What are the implications?
Line 259. And some NMD targets do not rely on UPF2 for NMD.
Line 263. CASC3 is "the fourth EJC constituent"?
Line 268. "CASC's primary function is in NMD" is just a guess.
Line 279. This is neither the sole nor first evidence that NMD depends on translation.
Line 291. The meaning of "UPF2 promotes expression...but promoting its local translation" is very unclear.
Line 392. The significance of "lacking SMG6 and the NMD-associated protein TDRD6' is unclear.
Line 395. Kebaara and Atkin is a study of yeast and should be removed.

Any additional non-essential suggestions for improving the study (which will be at the author's/editor's discretion)

This reviewer didn't make it to the end of the manuscript. Please start from the beginning and be sure each sentence has the appropriate meaning.

Referee #3:

Tan and Wilkinson present an excellent, comprehensive, critical, and accessible review of the functions of nonsense-mediated RNA decay (NMD). This pathway, which detects mRNAs with truncated open reading frames, has increasingly been recognized not only as a key mechanism of cellular quality control but also as an important regulator of fundamental biological processes. These include early embryonic development, the development and function of the nervous system, hematopoietic system development, and germ cell maturation, particularly spermatogenesis. In their review, the authors synthesize important experimental and clinical findings and provide a thoughtful appraisal of current uncertainties regarding the functions of NMD co-factors, both in NMD sensu strictu and in secondary or independent biological pathways.

Overall, this review effectively integrates important data and discusses the biological implications of NMD, covering a spectrum from RNA quality control to developmental and clinical contexts.

The manuscript could be further strengthened by including a dedicated section on both previously unsuccessful and current strategies aimed at modulating NMD efficiency. In particular, the authors could elaborate on how NMD inhibition may be therapeutically beneficial in specific genetic diseases-beyond the brief mentions of Duchenne muscular dystrophy and cystic fibrosis-and discuss approaches to target acquired diseases such as cancers with mismatch repair deficiency.

Finally, for a more balanced assessment of the literature, it would be valuable to include the work of Niels Gehring et al. when discussing the role of exon junction complex (EJC) components in activating alternative NMD pathways (PMID: 16209946), as well as the study by Thermann et al. on the importance of premature termination codon (PTC) position relative to downstream splice sites (PMID: 9628884).

Referee #1:

This review starts with a brief but sufficient overview of the basis of NMD, followed by a detailed review of work on mouse models and stem cell studies. The review reinforces the branched nature of NMD and reflects on its roles in embryonic development, neural development and signalling, different steps in spermatogenesis, hematopoietic cell proliferation, liver homeostasis and the circadian rhythm. The review ends with a consideration of future work to address the specific roles and molecular mechanisms of NMD, and the feasibility of NMD modulation therapy. It is engagingly written and reflects an extensive review on the impact of NMD developmental and cell differentiation pathways in mammalian systems. I only have a few minor comments that are listed below.

***Response**: We thank Reviewer 1 (Rev 1) for their positive comments about our review. Below, we answer all of Rev 1's critiques and indicate how we edited our manuscript accordingly.*

There are few grammar errors:

Line 224 To define the underlying mechanism,...

***Response**: We have added "the" in this sentence (line 205).*

Line 247 I don't think the abbreviation NPC (neural progenitor cell?) is defined

***Response**: NPC (neural progenitor cell) was previously defined (line 66 of the revised manuscript).*

Line 235 The phrase OSN neurons is tautological, given OSN is the abbreviation of olfactory sensory neuron

***Response**: We have omitted "neuron" (line 215).*

Line 338 I don't think that the abbreviation MVH is explained.

***Response**: We have removed this point from the revised manuscript, as MVH is not known to be pertinent to NMD function and thus is unnecessary to bring it up here.*

Line 715 opposing views...

***Response**: We have changed "view" to "views" (line 709).*

line 738 strange phrasing: maybe compared with instead of than?

***Response**: We have revised this sentence to the following: "As further evidence for UPF3B being unique in its specificity, a recent study showed that UPF3B promotes the decay of a set of NMD-target mRNAs that only minimally overlaps with those targeted for decay by the core NMD factor, UPF2, in both human ESCs and NPCs (Tan et al., 2025)" (lines 731-733).*

line 760 similar strange phrase: by which instead of how ?

***Response**: We have revised the sentence to the following: "While there has been some progress in ascertaining the underlying molecular mechanisms by which NMD influences biological events in cultured cells, virtually nothing is known about this topic in vivo" (lines 52-753).*

There are cases where citations are embedded within hyphens (e.g. Notaras et al. line 284; Tan et al. line 356; Frishmeyer-Guerrerio et al. line 435; Chousal et al. line 523; Huth et al. line 562) (I may not have included all examples here), which seems unnecessary - why not simply have the embedded citation?

***Response**: We have corrected this.*

Some references appear to lack page numbers: lines 805, 849, 854, 867, 876, 947, 967, 1031, 1062, 1064, 1092

***Response**: These cited articles are from journals that use an article-number system instead of traditional pagination. Indeed, we have checked these references and confirmed they have no page numbers in the published version. We have retained the correct article numbers in accordance with the journal's citation style.*

Fig. 1 perhaps emphasise this relates to roles of NMD in mammalian cells?

***Response**: We have revised the title of Figure 1 to: "Some biological roles of NMD in mammals."*

Fig 4 This is an effective figure but it would be clearer to me if it were rearranged to show, from left to right, the disease (e.g. CF), the condition (e.g. nonsense mutations), the NMD therapy (e.g. inhibtion) and the effect (e.g. increased expression of disease gene). The middle section labelled "Condition" is actually the consequence of the treatment rather than condition of the disease state, which is confusing.

***Response**: We have revised Figure 4, as suggested.*

Table 1 The column headed Non-NMD functions includes both functions of NMD factors (e.g. DNA damage response) and substrates (?) (E3 ligase and PI3K family member).

***Response**: We thank Rev 1 for noting this. Neither "E3 ligase" nor "PI3K family" refer to NMD substrates, but nonetheless they are not clear non-NMD functions and so we have omitted them from Table 1.*

Referee #2:

General summary and opinion about the principal significance of the study, its questions and findings

Mechanistic details provided at the beginning of the paper detract from the strength of the paper. Many sentences there and throughout are misleading or incorrect. This review is on an important topic but requires re-writing for clarity, accuracy and the complete and balanced story (including references).

***Response**: We appreciate Rev 2's thorough review and suggestions. Below, we answer all of Rev 2's critiques, and indicate how we edited our manuscript accordingly.*

Specific major concerns essential to be addressed to support the conclusions

1. There are many examples of mechanistic details at the start of the paper that are misleading, incomplete or incorrect. Here is a partial list.

The first paragraph of the Introduction applies largely to yeast and should be clarified as such since what led to the "mini-field", as the authors call it, are the natural NMD targets in mammalian cells, which have no counterpart in yeast.

***Response**: The point of the first paragraph was to acknowledge the first studies done in the field on NMD. We agree it is important to make it clear that these studies were done in Saccharomyces cerevisiae (yeast), which have added to the second sentence: "A research group studying yeast (Saccharomyces cerevisiae) discovered that introduction of nonsense mutations into genes elicits rapid degradation of their mRNA products (Losson et al, 1979)" (lines 27-29).*

Third paragraph: Probably the first "normal" mRNA degraded by NMD that encodes a full-length protein was described much earlier (1998, on glutathione peroxidase mRNA) than those referenced. Also, the reference to LeLivelt and Culbertson is off-topic - on surveillance (see comment above).

***Response**: We have added the citation Moriarty et al. 1998. As for the Lelivelt & Culbertson, 1999 citation, this paper defines normal (not aberrant) yeast mRNAs regulated by NMD and thus we feel it is relevant. We have retained this citation.*

Line 52. UPF2 and UPF3 are now considered auxiliary NMD factors, unlike UPF1. The author essentially writes this when calling UPF3B an "NMD branch" factor. In this context, though, what is UPF3A?

***Response**: To our knowledge, UPF3A has never been defined as either a "NMD branch" or "auxiliary NMD factor." In the same paragraph in which we introduced the other UPFs, we introduced UPF3A as follows: "UPF3A (also known as "UPF3") is a very weak NMD factor in most cell types (with the possible exception of spermatocytes; see below) and thus there have been efforts to determine if UPF3A has other functions (Ma et al, 2019; Shum et al, 2016; Xie et al, 2023)" (lines 59-62). We provide a more detailed discussion of UPF3A in a later paragraph (on lines 156–172).*

Line 65. How has this notion been challenged?

***Response**: A recent study using genome-wide approaches showed that UPF3B promotes the degradation of many NMD-target RNAs distinct from those dependent on the core NMD factor UPF2 (Tan et al., 2025). This challenges the view of UPF3B is merely an NMD-branch factor and instead suggests that UPF3B has the ability to target unique mRNAs. Accordingly, we have revised the text to: "However, this notion was recently challenged by the finding that UPF3B largely targets different mRNAs for decay than does the core NMD factor, UPF2, in both embryonic stem cells (ESCs) and neural progenitor cells (NPCs) (Tan et al, 2025). This suggests that UPF3B has the ability to target unique mRNAs for decay and thus is not merely a NMD-branch factor" (lines 64-67).*

Lines 69-74. This is all true, but it does not do the EJC justice, omitting lots of important information. For example, while the "accessory protein" CASC3 is recruited to the EJC in the cytoplasmic, so is UPF2, which was previously defined as one of the three key NMD factors, but on a different EJC.

***Response**: We thank Rev 2 for pointing this out. We have added two sentences to this "EJC paragraph" that reads: "In addition, different EJC proteins can serve as entry points for the formation of distinguishable NMD-activating ribonucleoproteins. For example, Y14, MAGOH, and eIF4AIII can activate NMD in a UPF2-independent manner, whereas evidence suggests that RNPS1-dependent NMD requires UPF2 (Gehring et al., 2005, Mabin et al., 2018)" (lines 73-76).*

Line 87. How is this an exception to the "downstream exon-exon junction rule"?

***Response**: It is an exception in the sense that in-frame stop codons closer than 55 nt to the last exon-exon function typically do not elicit NMD. To provide a clearer overview of NMD-inducing signals, we have added a box (Box 1) to the revised manuscript that explains NMD-inducing signals. This box (lines 1140-1168) contains what was previously in the text, and also has some new content (to help clarify the concepts). The box reads as follows:*

"Early studies demonstrated that one signal that triggers RNA decay by NMD is the presence of an exon-exon junction downstream of the stop codon defining the end of the main ORF (Carter et al, 1996; Nagy et al, 1998; Thermann et al, 1998). Later studies demonstrated that the molecular entity responsible for this "dEJ rule" is the EJC (Chang et al., 2007; Kishor et al., 2019; Kurosaki et al., 2019). Given that EJCs are generally recruited to all exon-exon junctions, why do only exon-exon junctions downstream of the stop codons trigger NMD? The likely answer comes from a study that demonstrated that EJC protein, Y14, is displaced from RNAs by translocating ribosomes after RNAs are transported into the cytoplasm (Dostie et al, 2002). Thus, mRNAs with exon-exon junctions only in the coding region will be stripped of all EJCs by translocating ribosomes and thus avoid NMD. In contrast, RNAs that have one or more exon-exon junctions after the stop codon will retain EJCs since ribosomes cease translocating after reaching a stop codon. This scenario leads to a model in which mRNAs with at least one exon-exon junction downstream of the main ORF will harbor an NMD-promoting EJC complex for an extended period of time and thus are primed to undergo decay through NMD. In support, introduction of exon-exon junctions downstream of ORFs has been shown to trigger NMD (Carter et al., 1996; Nagy et al., 1998; Thermann et al., 1998), and a large fraction of NMD-target mRNAs have at least one exon-exon junction downstream of the main ORF (Kurosaki et al., 2016; Nagy et al., 1998). An exception to this dEJ rule is the -55 nucleotide (nt) boundary rule, a rule based on the finding that RNAs with the main ORF stop codon less than ~55 nt upstream of the last exon-exon junction often escape NMD (Nagy et al., 1998). A likely explanation for this -55 nt boundary rule is that stop codons at such positions allow translocating ribosomes to collide with the EJC just upstream of the last exon-exon junction and thus displace this EJC from the RNA (given the known EJC "footpad" size and the fact that the EJC tends to be centered ~20-24 nt upstream of exon-exon junctions (Le Hir et al., 2016)).

There are also non-EJC-dependent mechanisms that degrade RNAs by the NMD pathway. For example, long 3' untranslated regions (UTRs) and short ORFs upstream of the main ORF (uORFs) can trigger NMD (Kishor et al., 2019; Kurosaki et al., 2019; Munoz et al., 2023). While both long 3'UTRs and uORFs can elicit NMD, they do not necessarily do so. No specific 3'UTR length has been shown to elicit NMD (Toma et al, 2015). Half of mammalian mRNAs contain uORFs and thus most uORFs must not be capable of eliciting NMD. Even an exon-exon junction downstream of a stop codon—a dEJ—will not necessarily elicit NMD; this depends on biological context (Tan et al., 2025)."

Line 94. This paragraph is very confusing. First, branches are defined as NMD pathways that are either EJC-dependent or not, then uORFs are mentioned. It is unclear how the authors can consider branches as also defined by UPF3B, RNPS1 and CASC3, which returns to an EJC-dependent pathway, presumably. What about AKT?

***Response**: To avoid confusion, we have removed a detailed discussion of "NMD branches" from this review, as this is not the focus of our review. What we **do** cover is NMD-inducing features (e.g., dEJs, uORFs and long 3'UTRs; see new Box 1), as it is essential that readers understand the signals that trigger RNAs to be degraded by NMD. We note that the topic of NMD branches is covered well by Yi et al. 2021 (Trends Gen; PMID: 33008628), which we cite.*

2. Starting with line 106, there is insufficient information in the sentences to understand significance. E.g. what are "NMD-deficient Caenorhabditis elegans" and, considering the next sentence, do they have a shortened lifespan - how was NMD shown to increase lifespan?

***Response**: Rev 2 brings up good points. To address this, we have revised the sentences on this topic as follows: "In Caenorhabditis elegans, NMD has roles in mating. NMD-deficient C. elegans (harboring mutations in smg genes) have morphogenic defects in the male bursa and hermaphrodite vulva (Cali et al., 1999, Hodgkin et al., 1989). Interestingly, NMD has been shown to also influence C. elegans lifespan under some circumstances. Loss of NMD (as a result of knockout or knockdown of the smg-2 gene) substantially shortens the extended lifespan elicited by simulating starvation (via genetic inhibition of the daf-2/insulin/IGF-1 receptor pathway) (Son et al., 2017). Given that NMD activity declines during C. elegans aging (Son et al., 2017), these results have potential implications for the effects of diet and aging in humans" (lines 82-88).*

Minor concerns that should be addressed (incomplete list)

Line 178. I wouldn't think that a putative repressor of NMD is "a non-NMD function".

***Response**: Our intent was to convey that UPF3A has been found to be an extremely weak NMD factor in all cell types examined to date and thus its biological functions are not readily explained by its NMD activity alone. A particularly striking example of UPF3A's biological function is during early embryonic development. Loss of UPF3A causes early embryonic lethality, while loss of its paralog, UPF3B, is dispensable for embryonic development. Thus, we believe it is reasonable to hypothesize that UPF3A acts biologically (e.g., in early embryonic development and spermatogenesis) by a mechanism independent of promoting NMD. That said, we previously may have been too strident in proposing this idea. In the revision, we have softened the first sentence of the relevant paragraph to: "The NMD factor, UPF3A, is a good candidate to influence embryonic development through non-NMD biochemical functions" (lines 156-157).*

Line 190. It has been shown that TA can also upregulates the nonsense-containing gene. Also, "paralogs of gene" should be "paralogs of some genes".

***Response**: We apologize for the typo; we have revised the text to say "paralogs of some genes" (line 167). With regard to the other point, we are familiar with the evidence for this point, but we feel it is beyond the scope of our review to describe the transcriptional adaptation (TA) response to this level of detail. Furthermore, this review is already quite lengthy. That said, if deemed critical, we can add a sentence on this point.*

Line 211. Is it that NMD promotes cell proliferation or that the regulated inhibition of NMD promotes apoptosis?

***Response**: NMD has been shown to promote cell proliferation and/or survival (the latter by inhibiting apoptosis) in several different biological systems. We have revised the relevant section of the manuscript with the following: "Enriched among these NMD targets are functions related to cell-cycle arrest and apoptosis, raising the possibility that NMD acts in early mouse embryogenesis by promoting cell proliferation and/or cell survival. Indeed, it is known that NMD promotes cell proliferation and prevents programmed cell death in other biological contexts, as well as in species in addition to mice (Avery et al., 2011; Lou et al., 2014; Nelson et al., 2016; Weischenfeldt et al., 2008)." (lines 190-194).*

Line 233. Please specify how the UFP3B targets were identified.

***Response**: We have added the following clarification: "In these studies, high-confidence NMD-target mRNAs were identified based on (i) their being upregulated upon UPF3B loss and (ii) their containing at least one well-established NMD-inducing signal" (lines 215-217).*

Line 241. What are the implications?

***Response**: We have revised the end of the paragraph to say: "This unexpected finding suggests that NMD-target mRNAs can be highly context-dependent, varying across brain regions and possibly different neuronal cell types. This may contribute to distinct biological functions of NMD in different parts of the brain" (lines 221-224).*

Line 259. And some NMD targets do not rely on UPF2 for NMD.

***Response**: We have removed "including Upf2" from the sentence on line 241.*

Line 263. CASC3 is "the fourth EJC constituent"?

***Response**: We have revised as follows: "In this regard, it is worth noting that KO of the gene encoding the non-constitutive EJC subunit—CASC3—causes much less severe brain defects than KO of genes encoding the constitutive EJC components RMB8A, MAGOH, and EIF4A3 (Mao et al, 2017)" (lines 246-248).*

Line 268. "CASC's primary function is in NMD" is just a guess.

***Response**: We examined the literature more carefully and found evidence that CASC3 is involved in cytoplasmic RNA localization and translational regulation. Thus, we have revised the end of this paragraph to say: "It will be important in the future to determine whether this is because CASC3 differs from other EJC components in being primarily recruited to the EJC in the cytoplasm rather than in the nucleus (Mabin et al., 2018). While one of CASC3's functions is in NMD (Mabin et al., 2018), it also influences translation and cytoplasmic RNA localization (Chazal et al, 2013; Macchi et al, 2003; van Eeden et al, 2001)" (lines 249-253).*

Line 279. This is neither the sole nor first evidence that NMD depends on translation.

***Response**: We have added a key early paper demonstrating a role for translation in NMD: Belgrader et al, 1993.*

Line 291. The meaning of "UPF2 promotes expression...but promoting its local translation" is very unclear.

***Response**: We have revised this section to read as follows (lines 274-280): "This analysis revealed that UPF2 promotes expression of the excitatory neurotransmitter receptor, Glutamate Receptor 1 (GLUR1), by promoting its translation specifically in dendrites – the subcellular region where GLUR1 functions. These authors found that UPF2 also drives GLUR1 expression by another mechanism - it inhibits GLUR1 protein internalization, thereby promoting dendritic cell-surface expression. As evidence that UPF2 acts at the local level, these investigators found that UPF2, as well as other NMD factors, are concentrated in dendrites."*

Line 392. The significance of "lacking SMG6 and the NMD-associated protein TDRD6' is unclear.

***Response**: We have revised this sentence to read: "Similarly, Lehtiniemi et al. and Fanourgakis et al. found that dEJ-containing transcripts were not enriched in the upregulated transcripts in round spermatids made NMD deficient as a result conditional loss of the NMD protein, SMG6, or the NMD-associated protein, TDRD6, respectively (Fanourgakis et al., 2016; Lehtiniemi et al., 2022).*

Together, these findings suggest the possibility that male germ cells are unique in not recognizing the dEJ NMD-inducing signal" (lines 378-382).

Line 395. Kebaara and Atkin is a study of yeast and should be removed.

***Response**: We have done this.*

Any additional non-essential suggestions for improving the study (which will be at the author's/editor's discretion)

This reviewer didn't make it to the end of the manuscript. Please start from the beginning and be sure each sentence has the appropriate meaning.

***Response**: Each author has independently read through the entire manuscript to scrutinize for errors in clarity and other issues.*

Referee #3:

Tan and Wilkinson present an excellent, comprehensive, critical, and accessible review of the functions of nonsense-mediated RNA decay (NMD). This pathway, which detects mRNAs with truncated open reading frames, has increasingly been recognized not only as a key mechanism of cellular quality control but also as an important regulator of fundamental biological processes. These include early embryonic development, the development and function of the nervous system, hematopoietic system development, and germ cell maturation, particularly spermatogenesis. In their review, the authors synthesize important experimental and clinical findings and provide a thoughtful appraisal of current uncertainties regarding the functions of NMD co-factors, both in NMD sensu strictu and in secondary or independent biological pathways.

Overall, this review effectively integrates important data and discusses the biological implications of NMD, covering a spectrum from RNA quality control to developmental and clinical contexts.

***Response**: We thank Rev 3 for their positive comments about our review. Below, we answer all of Rev 3's critiques and indicate how we edited our manuscript accordingly.*

The manuscript could be further strengthened by including a dedicated section on both previously unsuccessful and current strategies aimed at modulating NMD efficiency. In particular, the authors could elaborate on how NMD inhibition may be therapeutically beneficial in specific genetic diseases-beyond the brief mentions of Duchenne muscular dystrophy and cystic fibrosis-and discuss approaches to target acquired diseases such as cancers with mismatch repair deficiency.

***Response**: We thank Rev 3 for the thoughtful suggestion to expand the discussion on therapeutic modulation of NMD, including its potential relevance to cancers with mismatch repair deficiency. We have added the following sentence: "NMD modulatory therapy may also have a role in treating cancer; e.g., by upregulating the expression of frameshift-derived immunogenic peptides from tumors with defective DNA mismatch repair (Becker et al, 2021; Tan et al., 2022a)" (lines 473-475). In addition, we now refer to a very recent review that provides an excellent summary of therapeutic NMD modulation: "More detailed information on future therapeutic NMD approaches can be found in a recent review (McMahon et al, 2025)" (lines 475-476).*

Finally, for a more balanced assessment of the literature, it would be valuable to include the work of Niels Gehring et al. when discussing the role of exon junction complex (EJC) components in activating alternative NMD pathways (PMID: 16209946), as well as the study by Thermann et al. on the importance of premature termination codon (PTC) position relative to downstream splice sites (PMID: 9628884).

***Response**: We thank Rev 3 for highlighting these important studies. In the revised manuscript, we have incorporated the following sentence to reflect the findings of the Gehring et al. paper: "In addition, different EJC proteins can serve as entry points for the formation of distinguishable NMD-activating messenger ribonucleoproteins. For example, Y14, MAGOH, and eIF4AIII can activate NMD in a UPF2-independent manner, whereas evidence suggests that RNPS1-induced NMD requires UPF2 (Gehring et al, 2005; Mabin et al, 2018)" (lines 73-76). We have also cited Thermann et al. in the new Box 1.*

Dear Prof. Wilkinson,

Thank you very much for re-submitting your review proposal (EMBOJ-2025-121891R) to The EMBO Journal. The manuscript has now been re-evaluated by one of the original three referees, who finds the revised version to be improved and recommends publication. The remaining referees did not respond or were unable to re-review the manuscript, and we therefore decided to proceed based on the positive evaluation from referee #3.

I have also read this revised version of the review proposal and agree with the referee that the manuscript is well structured and provides a clear summary and analysis of the recent literature. That said, I believe that some sections-particularly parts of the introduction-would benefit from language editing, especially the first paragraph and some of the text added during the first round of revisions. In addition, I think it would be beneficial to emphasize the review's focus on mammalian biology in the title.

Most importantly, the manuscript would benefit from further work on the figures, both in terms of the level of detail of the information presented and their overall visual cohesiveness. We would be happy to assist with this once the content of the figures has been finalized. Finally, there is one formal editorial request to remove the author contribution section from the manuscript file. I would be happy to discuss any of these points with you by email should you have any questions.

I apologize for the lengthy delay in my assessment of the manuscript, which was due to the unavailability and delayed response of one of the referees, as well as a medical condition that prevented me from working during the holiday period.
With best regards,

Cornelius Schneider

Cornelius Schneider, PhD
Editor
The EMBO Journal
c.schneider@embojournal.org

Referee #3:

The authors have comprehensively addressed my previous comments and I recommend publication

The authors have addressed all minor editorial requests.

2nd Revision - Editorial Decision 10th Apr 2026

Dear Prof. Wilkinson,

I am pleased to inform you that your manuscript has been accepted for publication in the EMBO Journal.

Your manuscript will be processed for publication by EMBO Press. It will be copy edited and you will receive page proofs prior to publication. Please note that you will be contacted by Springer Nature Author Services to complete licensing and payment information.

You may qualify for financial assistance for your publication charges - either via a Springer Nature fully open access agreement or an EMBO initiative. Check your eligibility: https://link.springer.com/journal/44318/how-to-publish-with-us

Should you be planning a Press Release on your article, please get in contact with embo_production@springernature.com as early as possible in order to coordinate publication and release dates.

If you have any questions, please do not hesitate to contact the Editorial Office. Thank you for your contribution to The EMBO Journal.

Yours sincerely,

Cornelius Schneider, PhD
Editor
The EMBO Journal
c.schneider@embojournal.org

*****
Please note that it is The EMBO Journal policy for the transcript of the editorial process (containing referee reports and your response letters) to be published as an online supplement to each paper. If you should prefer removal of any referee-only figures included in the point-by-point response(s), e.g. because they may still be used for future publication or because they have been reproduced from published work by others, please do let us know immediately via response email.
More information is available here: https://link.springer.com/partners/embo-press/editorial-policies#Peer%20review
